# Dual Targeting Strategies in Cancer: Carbonic Anhydrase IX Inhibitors Targeting EGFR or VEGFR-2

**DOI:** 10.3390/molecules31132306

**Published:** 2026-07-01

**Authors:** Eleftherios Charissopoulos, Eleni Pontiki

**Affiliations:** Laboratory of Pharmaceutical Chemistry, School of Pharmacy, Faculty of Health Sciences, Aristotle University of Thessaloniki, 54124 Thessaloniki, Greece; echarise@pharm.auth.gr

**Keywords:** cancer, carbonic anhydrase, VEGFR-2, EGFR, multitarget

## Abstract

Tumor microenvironment influences the process of tumorigenesis, with hypoxia being a characteristic of many solid tumors and an adverse prognostic factor. Carbonic anhydrases (CAs) are highly efficient zinc-containing enzymes that are overexpressed in many cancers, particularly under acidic and hypoxic conditions. CA IX expression promotes cancer cell proliferation, migration, and invasion. Vascular endothelial growth factor receptor-2 (VEGFR-2) is a tyrosine transmembrane (ΤΜ) protein regulating embryonic development, angiogenesis, tissue homeostasis and cancer. Blocking VEGFR-2 signaling is one of the most promising approaches to hindering angiogenesis and growth of cancer cells. The epidermal growth factor receptor (EGFR) is a member of the ERBB family of receptor tyrosine kinases and plays a key role in cancer progression. EGFR is uniquely found in some brain, lung and other cancers. Development of novel strategies to regulate these factors is important for the treatment of tumors. Multifunctional drugs that act on multiple pathways offer a promising approach, improving therapeutic effectiveness while reducing side effects. The present review focuses on novel compounds that inhibit CA IX and target VEGFR-2 or EGFR.

## 1. Introduction

### 1.1. Cancer Progression: Hypoxia, Survival, and Angiogenesis

**Figure 1:**
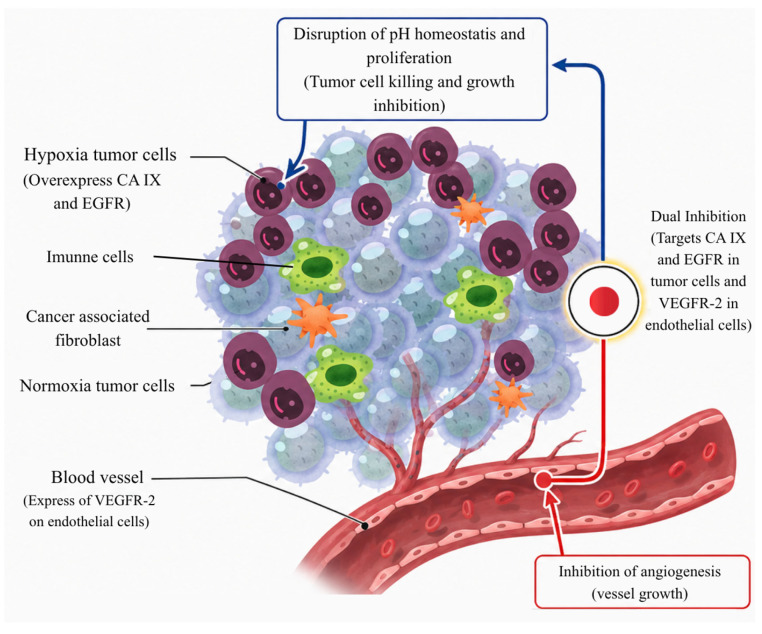
Schematic representation of the tumor microenvironment and the multi-target effects of dual CAIX/kinase inhibitors.

Cancer is a type of tumor characterized by abnormal cell growth that can invade or spread from its original organ to other parts of the body [1]. Cancer is considered to have enigmatic and complex features, which represent significant obstacles for both researchers and healthcare professionals in finding potential therapeutic strategies [2]. Mutations in genes encoding cell cycle regulatory proteins are primary drivers of carcinogenesis. These defective genes can be inherited or may arise in response to environmental carcinogens [3]. Tumor angiogenesis contributes to the survival and development of tumor cells, and plays a key role in their growth, invasion, and metastasis [4]. Various distinct biological processes occur in tumor vascularization, depending on the tumor type and anatomic location. These processes are regulated by a range of secreted factors and signaling pathways involving non-endothelial cells, such as progenitors or cancer stem cells [5]. Apoptosis is a regulated and evolutionarily conserved cell death program, and cancer cells often shows resistance to apoptosis, endowing them with survival advantages that promote tumor evolution, outgrowth and treatment failure [6]. Moreover, tumor cells can adapt to changes in oxygen tension which gives them ability to develop. Hypoxia is a critical microenvironmental factor, which defines a tumor’s growth and aggressiveness (Figure 1). At low oxygen concentration, cancer cells induce glycolytic metabolism, providing them with significant advantage [7,8]. The lack of novel multitarget drugs combined with the incomplete understanding of the molecular mechanism, is a main obstacle in metastasis prevention and treatment.

### 1.2. Carbonic Anhydrase IX: Biological Characteristics and Role in Cancer

#### 1.2.1. Carbonic Anhydrase Isoforms and Structural Features of CAIX

CAs are metalloenzymes catalyzing the reversible hydration of carbon dioxide to bicarbonates and protons [9,10]. More than sixteen isoforms of CAs, which includes human isoforms, have been reported to show a variety of activity profiles in humans, and the expression of some isoforms is disease-specific [11]. Only twelve of the human isoforms are catalytically active and the others do not show enzymatic activity [9]. Isoforms differ in location, quantity and function in the body, leading to differences in the structure, pharmacological activities and clinical effects of their inhibitors [12]. These isoforms are classified according to where they are located within cells: cytosolic (CA I, CA II, CA III, CA VII, and CA XIII), membrane-bound (CA IV, CA IX, CA XII, CA XIV, and CA XV), secreted (CA VI), and mitochondrial (CA VA and CA VB) [9]. CAIX is a highly active TM hypoxia-induced metalloenzyme and is mainly overexpressed in overwhelming majority of clear cell renal cell carcinoma [13,14]. Due to its low expression in a few sites in healthy tissues, it is considered a potent target for cancer therapy [14,15]. CAIX protein contains an extracellular-facing catalytic domain tethered to the plasma membrane by a single TM domain and a short intracellular (IC) *C*-terminal tail. In addition, CAIX possesses a unique *N*-terminal proteoglycan-like domain (PG domain) in comparison to the other isoforms [16,17,18].

#### 1.2.2. Regulation and Functional Role of CAIX in Cancer

**Figure 2:**
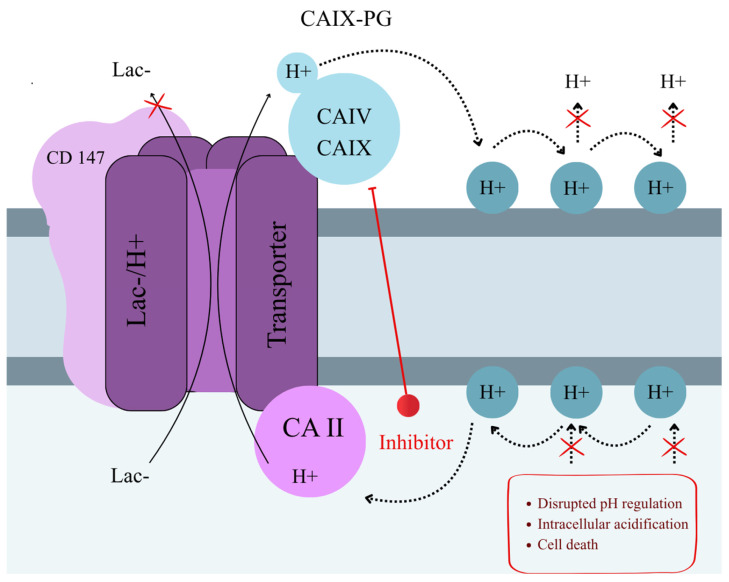
Targeting tumor cell membrane dynamics: CA IX and acid transport.

The expression of CAIX is controlled by the HIF transcriptional activity [19]. Hypoxia inducible factor (HIF) plays a crucial role in regulating the hypoxic-induced stress response in tumor and normal cells. An increase of HIF-1 is related to activating hypoxia-inducible genes that express hypoxia-responsive elements (HRE) [20]. It is observed that not only hypoxia, but also other microenvironment conditions, signaling pathways, soluble factors and genome mutations can regulate CA IX expression [21]. CAs and other transport proteins cooperate to regulate acid/base homeostasis in cancer cells. These proteins either promote proton extrusion or the movement of acid/base equivalents, such as lactate and bicarbonate, across the cell membrane [16]. As an essential part of a transport metabolon, it participates in the acidification of the extracellular space and the maintenance of IC pH within physiological ranges (Figure 2) [13]. At the exofacial location of the plasma membrane, CAIX catalyzes the reversible hydration of CO_2_ [15,22]. By turning CO_2_ into HCO_3_^−^ and H^+^, CAIX promotes CO_2_ removal, acidifies the outside of the cell, and keeps the IC pH more alkaline [23]. Changes in pH can affect tumor metastasis by splicing certain key proteins. These protein alterations contribute to increased aggressiveness, enabling tumor cells to breach the surrounding extracellular matrix (ECM) [12]. By directly binding to β-catenin and disrupting the E-cadherin/cytoskeleton/β-catenin complex, CAIX expression promotes cancer cell proliferation, migration, and invasion [19]. Sulfonamides have been the most widely developed class of inhibitors targeting CA IX over the past years. Several sulfonamides have already been synthesized (Table 1), possessing strong inhibitory activity, but with a limitation in their selectivity [24,25]. Despite the strong biological rationale for targeting CA IX in hypoxic tumors, its translation into successful clinical monotherapies has progressed slowly. This is due to the limited structural conservation across the 15 human CA isoforms, where off-target inhibition of the ubiquitous cytosolic isoforms CA I and CA II can lead to systemic toxicities like metabolic acidosis [21].

### 1.3. EGFR: Structure, Activation and Role in Cancer

EGFR belongs to the ERBB family of tyrosine kinase (TK) and plays an important role in the cancer progression [29]. EGFR and family members are reported to be involved in numerous diseases like inflammation, psoriasis, cardiovascular diseases, and cancer [30]. Four members of the receptor family constitute the EGFR family: ErbB1, ErbB2, ErbB3, and ErbB4, differing in their binding interactions with ligands [31]. Activation of EGFR pathway provides a strong signal for epithelial cell proliferation and survival during organogenesis and tissue repair [32]. In cancer, EGFR results in increased cellular proliferation with decreased apoptosis, thereby promoting tumor growth. EGFR consists of an extracellular ligand-binding site with a dimerization arm, a TM hydrophobic membrane and an IC tyrosine kinase and a *C*-terminal tail [30]. Normally, EGFR is a monomer located on the cell surface in a structurally compact autoinhibited state, displaying minimal kinase activity. When ligands such as epidermal growth factor (EGF), transforming growth factor alpha (TGFα), epiregulin (EREG), betacellulin, heparin-binding EGF-like growth factor (HB-EGF), amphiregulin (AREG), epigen, heregulin, and neuregulins 1–4 bind to the receptor, it results in receptor homo- or hetero-dimerization. which triggers phosphorylation of the tyrosine kinase domain [32,33]. This triggers signal transduction via the RAS-RAF-MAPK pathway, driving tumor growth and progression [33,34]. Due to the crucial role in regulation and activating key cellular processes, the development of molecular agents targeting the EGFR signaling pathway is a potent approach for designing promising anti-cancer drugs. Uncommon EGFR mutations represent a rare subgroup of non–small cell lung cancer (NSCLC). EGFR driver mutations are mainly located within the kinase domain, spanning exons 18–21, and lead to continuous activation of EGFR signaling pathways, resulting in uncontrolled cell growth and tumor development. While this treatment has benefited many patients with activating EGFR mutations, almost all who initially respond will eventually develop resistance. Approximately 50% of cases of acquired resistance are due to a secondary T790M mutation in exon 20 of the EGFR gene; however, many of the remaining resistance mechanisms are still unknown [35,36]. Osimertinib was an approved drug to treat T790M-positive patients who have advanced EGFR mutations after first- or second-generation EGFR TKIs. The high clinical efficiency of osimertinib was compromised by the occurrence of the C797S mutation. This mutation affects the key cysteine residue that forms covalent bonds with irreversible inhibitors, causing drug candidates to fail in achieving therapeutic efficacy [37].

### 1.4. VEGFR-2: Signaling and Inhibition in Tumor Angiogenesis

VEGFR-2 is an important tyrosine TM protein. There are three main VEGF receptors: VEGFR-1, VEGFR-2, and VEGFR-3. VEGFR-1 and VEGFR-2 are responsible for angiogenesis and vascular permeability, whereas VEGFR-3 primarily controls lymphangiogenesis. VEGFR-2 is mainly expressed in vascular endothelial cells and serves as the most potent signal transducer for angiogenesis [38]. The VEGF family is composed of VEGF-A, VEGF-B, VEGF-C, VEGF-D, and placental growth factor (PlGF) [39]. VEGFs are one of the most specific and crucial regulators of angiogenesis [40]. VEGF-A interacts with VEGFR-2 regulating endothelial cell proliferation, migration, angiogenesis and other biological functions [41,42]. VEGFR-2 is composed of three domains: the extracellular (EC), a single-pass α-helical TM and the IC containing two tyrosine kinase domains [43]. A kinase-insert domain separates the two tyrosine kinase domains. Binding of VEGF induces dimerization of two VEGFR-2 monomers, leading to their autophosphorylation [44]. The phospholipase-Cγ-protein kinase-C pathway is used by activated VEGFR-2 to activate MAP-kinase and DNA synthesis, and this leads to pathological angiogenesis. Src kinases, focal adhesion kinase, PI3K-PKB-Akt, Rho family of GTPases, are some reported VEGFR-2 dependent pathways [45]. Consequently, inhibition of VEGF/VEGFR-2 signaling pathway is a promising target for inhibition of tumor angiogenesis and subsequent tumor growth [46]. One target is to prevent the activation of VEGFR-2 receptors by using tyrosine kinase inhibitors (RTKIs) [40]. Sunitinib, sorafenib, lenvatinib, regorafenib and fruquintinib are some inhibitors that received USFDA approval for various types of cancer (Table 2). There are two types of kinase inhibitors based on how they target VEGFR-2. Type I inhibitors bind the active VEGFR-2 conformation around the ATP adenine-binding site. Type II kinase inhibitors stabilize the inactive DFG-out conformation, allowing them to bind a hydrophobic allosteric site next to the ATP pocket [38,40].

## 2. Dual CA IX and VEGFR-2 Inhibitors

### 2.1. Quinazoline-Sulfonamide Hybrids

**Figure 3:**
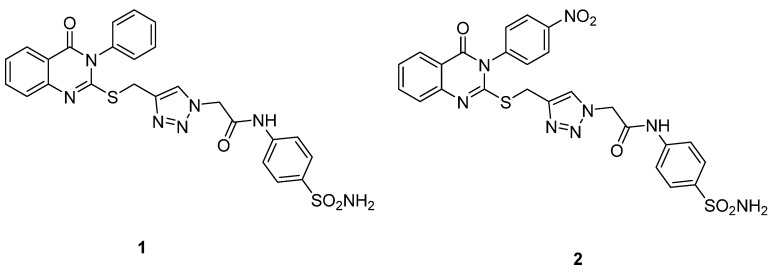
Chemical structures of enaminone-linked benzofuran compounds (**1**, **2**) [52].

Zeidan, M.A. et al. (2026) [52], synthesized a novel series of quinazoline-sulfonamide hybrids. The quinazoline and primary sulfonamide scaffolds were selected because they possess complementary anticancer properties. Primary sulfonamides are established CA inhibitors that efficiently coordinate the active-site Zn^2+^ ion, while quinazolines are privileged heterocyclic motifs widely used in anticancer drug design, particularly as kinase inhibitors. Compounds **1** and **2** (Figure 3) demonstrated strong multitarget inhibitory activity, exhibiting IC_50_ values of 0.145 and 0.069 μM against VEGFR-2, 0.168 and 0.077 μM against CA IX, and 0.154 and 0.205 μM against CA XII, respectively (Table 3). Their inhibitory potencies were comparable to those of the reference drugs sorafenib and acetazolamide (AAZ). Compounds **1** and **2** were systematically evaluated for their in vitro cytotoxic activity against a panel of human cancer cell lines, including HeLa, HepG2, HCT-116, and MCF-7, using the MTT assay. Compounds **1** and **2** demonstrated broad-spectrum cytotoxicity against HeLa, HepG2, HCT-116, and MCF-7 cell lines (IC_50_ = 11.57–21.43 μM for compound **1**; 7.81–19.50 μM for compound **2**) (Table 4). Compound **2** was found to induce G2/M phase arrest and trigger apoptosis in 47.25% of cells, accompanied by a sevenfold elevation in the Bax/Bcl-2 ratio and significant activation of caspase-3 and caspase-9. Molecular docking and dynamics studies have shown favorable multitarget binding, validating the proposed hybrid design strategy. In summary, the structure activity relationship (SAR) reveals that replacing a hydrogen atom (compound **1**) with a nitro group (compound **2**) significantly optimizes dual CA IX and VEGFR-2 potency. This marked reduction in values is driven by the strong electron-withdrawing properties and high dipole moment of the nitro moiety, which enhance binding affinity through distinct mechanisms. Ultimately, these findings demonstrate that the nitro substitution serves as an essential electronic driver rather than a passive steric modification, providing a valuable chemical blueprint for the future design of dual antiangiogenic and pH-disrupting antitumor agents.

### 2.2. Enaminone-Linked Benzofuran Derivatives

**Figure 4:**
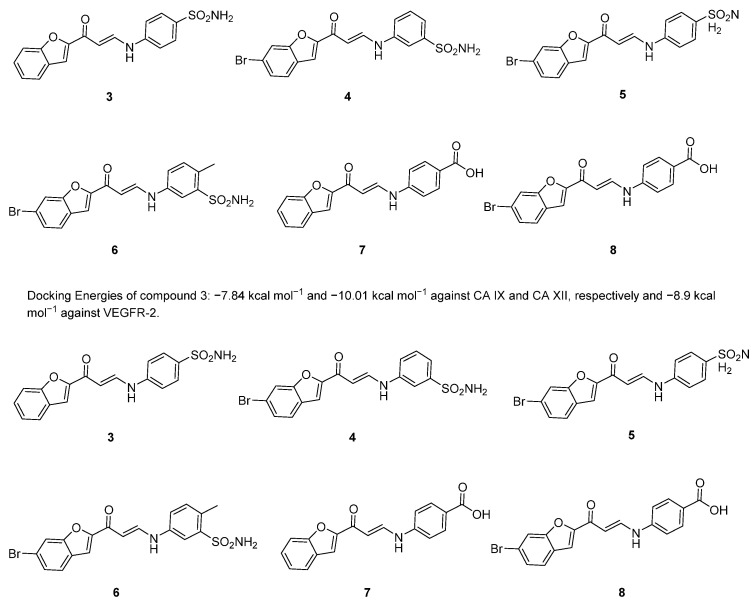
Chemical structures of enaminone-linked benzofuran compounds (**3**–**8**). Docking energies of compound **3**: −7.84 kcal mol^−1^ and −10.01 kcal mol^−1^ against CA IX and CA XII, respectively and −8.9 kcal mol^−1^ against VEGFR-2 [53].

Eldehna, W.M. et al. (2025) [53] synthesized and bioevaluated a novel series of enaminone-linked benzofuran compounds. The enaminone linker was chosen for its conjugation, flexibility, and ability to properly position zinc-binding groups for CA inhibition. The benzofuran core was selected for its anticancer potential and bioisosteric suitability for multitarget activity. Compounds were tested for their inhibitory activity against hCA I (cytosolic isoform) and the TM cancer-related isoforms hCA IX and hCA XII using a stopped-flow CO_2_ hydrase assay. AAZ was used as reference. Compound **3** (Figure 4) showed significant inhibitory effect against hCA IX with K_I_ = 35 nM compared to acetazolamide with K_I_ = 25 nM. Compounds **4**–**8** exhibited moderate inhibition, with K_I_ values 137, 171, 881.2, 882.2 and 754.2 nM respectively. The inhibitory activity against hCA I was quite mild, with K_I_s ranging from 6712 nM to over 100 μM. The para-benzenesulfonamide compound **3**, which had a K_I_ value of 6712 nM is the best homologue of this group, containing an unsubstituted benzofuran moiety. AAZ was the reference compound with K_I_ = 250 nM. Compounds were tested for their inhibitory effect against human carbonic anhydrase XII (hCA XII) and compound **3** was the only one having potent results, with a K_I_ value of 1.3 nM, compared to AAZ (5.7 nM). Selectivity for cancer-related isoforms (IX and XII) over the physiological isoform (I) was evaluated, and the results demonstrated preferential activity toward the cancer-associated isoforms. Selectivity ratio (SR) values were calculated by dividing K_I_ for physiological isoform by those for cancer-related isoforms. Compound **3** was the most selective of the sulfonamide group with a remarkable SR of 191.8 for hCAI/IX and an outstanding SR of 5163 for hCAI/XII, compared to AAZ with SRI/IX = 10 and SRI/XII = 43.8. Compound **4** (SR_I/IX_ = 382.9 and SR_I/XII_ = 237.3), showed potent selectivity, indicating the beneficial effect of the meta-positioned, unrestricted -SO_2_NH_2_ group on isoform selectivity. The most selective compounds for the tumor-associated isoforms hCA IX and XII contained non-hindered aromatic sulfonamide or carboxylic acid groups. The selectivity profile of compounds **3**–**5** was further assessed by including inhibitory data against hCA II, a ubiquitously expressed isoform. Compound **3** exhibited moderate activity toward hCA II (K_I_ = 7.5 nM). Compound **3** had ratios of SR_II/IX_ = 0.21 and SR_II/XII_ = 5.7. Compound **4** demonstrated better selectivity toward hCA IX and XII over hCA II (K_I_ = 980.8 nM for hCA II; SR_II/IX_ = 7.15, SR_II/XII_ = 4.43). Compound **3** showed balanced inhibition with moderate selectivity (K_I_ = 81.8 nM for hCA II; SR_II/IX_ = 0.47, SR_II/XII_ = 0.78). AAZ displayed relatively lower selectivity over hCA II against hCA IX and XII (K_I_ = 12.0 nM for hCA II; SR_II/IX_ = 0.48, SR_II/XII_ = 2.1). Overall, compound **1** showed strong inhibitory activity against hCA IX (K_I_ = 35.0 nM) and especially hCA XII (K_I_ = 1.3 nM). Compound **3** demonstrated much higher selectivity for the tumor-associated isoforms than acetazolamide, indicating that it is both potent and highly selective for hCA IX and XII over hCA I. Compounds **3**–**5** were selected for evaluation of their inhibitory activity on hypoxia-induced VEGFR-2, in vitro. Pazopanib (PAZ) was used as standard with IC_50_ = 0.059 μM. Compound **3** showed excellent inhibitory effect with IC_50_ = 0.058 ± 0.001 μM. Compound **5** exhibited increased inhibitory potency (IC_50_ = 0.155 ± 0.010 μM) in contrast to the meta-substituted compound **4**, which had a noticeably weaker impact (IC_50_ = 1.024 ± 0.220 μM) (Table 5). Compounds **3**–**5** were tested for their in vitro cytotoxic activity against MCF-7 and PC-3 cells in both normoxic and hypoxic conditions, through the MTT assay. DOX was used as a reference drug. The results indicate that all three compounds exhibited higher anticancer activity under hypoxic conditions than under normoxia, in contrast to the reference treatment DOX. Compound **3**, containing an unsubstituted benzofuran moiety (IC_50_ values of 9.03 μM against MCF-7 cells and 14.09 μM against PC-3 cells) and compound **5** (IC_50_ values of 3.65 μM against MCF-7 cells and 5.62 μM against PC-3 cells), containing a 5-bromo-substituted benzofuran moiety demonstrated greater cytotoxic activity under hypoxic conditions. In contrast, shifting the sulfonamide moiety in compound **4** from the *para* to the *meta* position led to a significant decrease in activity, with IC_50_ values of 17.56 μM (MCF-7) and 34.48 μM (PC-3). The results suggest that the combination of a *para*-positioned benzenesulfonamide group in compound **3** and an unsubstituted benzofuran ring plays a key role in improving cytotoxic activity in hypoxic environments. Normal human MCF-10A cells were utilized as a non-cancerous control to determine the selectivity index (SI) of Compound **3** in comparison to MCF-7 breast cancer cells. Safety and selective toxicity for cancer cells of compound **3** were supported by its weak activity against MCF-10A cells (IC_50_ = 41.36 ± 1.90 μM) (Table 6). The cytotoxic activity of compound **3**, both by itself and in conjunction with DOX, was assessed using the MTT test. The IC_50_ values for DOX increased significantly (up to 9.42 μM in MCF-7 and 6.06 μM in PC-3), indicating a hypoxia-induced chemoresistant phenotype. Under hypoxic conditions, DOX also lost much of its activity. Co-treatment with compound **3** reduced the IC_50_ of DOX from 9.42 to 2.15 μM in MCF-7 cells and from 6.06 to 2.94 μM in PC-3 cells. Results indicate that compound **3** can effectively overcome hypoxia-induced chemoresistance by restoring sensitivity to DOX in breast MCF-7 cancer cells. Compound **3** primarily induces cell-cycle arrest MCF-7 cells at the S phase. Annexin V-FITC/propidium iodide (PI) assay was conducted to evaluate apoptosis. Results showed that compound **3** mostly induces apoptosis rather than necrosis in MCF-7 cells, with early apoptosis being the most common kind of cell death. Compound **3** was tested for its effects on key apoptotic markers, including p53, caspase-9, Bcl-2, and BAX, in MCF-7 cells. Data expressed as fold change in gene expression showed that mRNA levels of BAX, p53, and caspase-9 were all significantly higher (2.98 ± 0.43, 5.72 ± 0.94 and 5.61 ± 0.58, respectively) when compared to the untreated control (1.00 ± 0.04, 1.00 ± 0.01 and 1.00 ± 0.09, respectively). Only Bcl-2 was significantly downregulated, dropping to a 0.55-fold change (± 0.06) with compound **3** treatment compared to the untreated control (1.00 ± 0.04). Compound **3** was assessed for its impact on MCF-7 cell migration using the wound-healing (scratch) assay. Untreated control cells, after 48 h, showed high rate of migration and wound closure, at 69.06%. Treatment with compound **3**, at IC_50_ 0.1 and IC_50_ 0.5 doses, significantly inhibited cell migration, resulting in reduced wound closure of 64.47% and 36.12%, respectively, after 48 h. Molecular docking studies were carried out against CA IX and CA XII of compound **3**. Compound **3** exhibited binding affinity values of −7.84 kcal mol^−1^ and −10.01 kcal mol^−1^ against CA IX and CA XII, respectively. Also, docking results showed that compound **3** successfully achieved the essential coordination with the catalytic Zn^2+^ ion through its sulfonamide moiety in both CA IX and CA XII active sites. Furthermore, molecular docking studies were conducted for VEGFR-2 in its active site to test the best binding mode. Compound **3** showed a better scoring function of a value of −8.9 kcal mol^−1^, suggesting its binding potential to VEGFR-2. ADMETlab 3.0 was used to estimate the ADMET profiles of compounds **3**–**5** and PAZ and results showed acceptable or ideal ranges, indicating balanced physicochemical and pharmacokinetic properties. Compound **3**, with an unsubstituted benzofuran group, was the most potent dual inhibitor, indicating that substitution on the benzofuran ring reduces activity. The para-positioned benzenesulfonamide moiety enhanced inhibition by favoring CA IX inhibition. Compound **3** exhibited IC_50_ values against CA IX and VEGFR-2 that were comparable to, and in some cases slightly lower than, those of the reference drugs, suggesting its potential as a dual inhibitor of both targets. Overall, this substitution pattern appears crucial for dual hCA IX and VEGFR-2 activity, making compound **3** a promising lead compound with noteworthy antimigratory and antiproliferative properties as well as dual target potential.

### 2.3. Isatin Hydrazone/1,2,3-Triazole Derivatives

**Figure 5:**
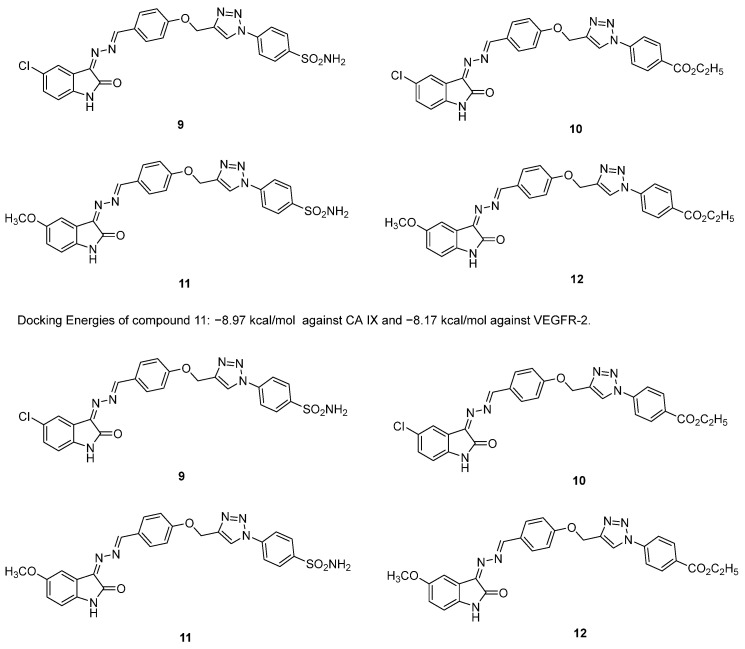
Chemical structures of isatin hydrazone/1,2,3-triazole compounds (**9**–**12**). Docking energies of compound **11**: −8.97 kcal/mol against CA IX and −8.17 kcal/mol against VEGFR-2 [54].

El-Attar, M.A.Z. et al. (2025) [54] developed and analyzed a series of isatin hydrazone/1,2,3-triazole hybrids. The isatin scaffold was converted into hydrazone–triazole hybrids to achieve structural flexibility and dual-target activity. Sulfonamide and ethyl ester groups were selected as zinc-binding motifs to enhance CA inhibition. Also, methoxy and chloro substituents at the isatin 5-position were used to modulate electronic properties for VEGFR-2 activity. Overall, the modifications were guided by known indolinone-based VEGFR-2 inhibitors to support balanced CA IX and VEGFR-2 dual inhibition. Inhibitory potential against the tumor-related human (h) CA iso forms IX, and XII, was evaluated in vitro, using a stopped-flow CO_2_ hydration assay. Also, cytosolic hCA isoforms I and II were evaluated. AAZ was the reference compound. All tested compounds showed weak or zero inhibition of hCA I isoform with K_I_ = 5573 to more than 10,000 nM, indicating promising isoform selectivity profile. Considering the cytosolic hCA II isoform, compounds **9** and **11** (Figure 5), having a 4-sulfonamide moiety, showed moderate inhibition (K_I_ = 182.60 ± 7.30 and 292.80 ± 10.50 nM, respectively) compared to AAZ (K_I_ = 12.10 ± 0.48). Compounds **9**, **11**, featuring a 4-sulfonamide moiety, and **12** exhibited low range inhibition against tumor-associated hCA IX. Compound **9** demonstrated the most potent inhibition of hCA IX with K_I_ value of 27.30 ± 1.10 nM, compared to reference compound (K_I_ = 25.80 ± 1.02 nM). Compound **11** showed K_I_ value of 31.50 ± 1.10 nM, trailing slightly behind was the 4-carbethoxy compound **12** with K_I_ value of 37.20 ± 1.50 nM. It appears that 4-sulfonamide moiety plays a crucial role in hCA IX inhibition, and chloro substitution enhances activity compared to the methoxy group. Among the tested compounds, only compound **8**, with a carbethoxy group at position-4 of the phenyl ring and chloro group on the 2-oxoindoline scaffold, displayed the strongest inhibition against hCA XII (K_I_ = 72.90 ± 2.80 nM, compared to AAZ with K_I_ = 5.70 ± 0.22 nM). Compounds **9**, **11** and **12** exhibited moderate inhibition with K_I_ values of 221 ± 8.40, 386.90 ± 12.10 and 726.90 ± 14.10 nM, respectively. Selectivity indices (SI) were calculated as the ratio of K_I_ values for the ubiquitous isoform hCA II to those for the tumor-associated isoforms (hCA IX or hCA XII). Compound **12** was approximately 23-fold more selective for hCA IX over hCA II. In addition, compounds **11** and **9** displayed favorable selectivity towards hCA IX (SI = 9 and 6.7-fold, respectively). Compound **10** exhibited significant selectivity toward hCA XII (SI = 8 and 9.7, respectively). Synthesized compounds were evaluated for their VEGFR-2 inhibitory activity. Sunitinib was the reference compound (IC_50_ = 0.09 ± 0.002 μΜ) (Table 7). All synthesized compounds exhibited potent inhibitory activity with compound **7** (IC_50_ = 0.06 ± 0.004 μΜ) having the best one. Compound **11** presented good inhibitory activity with IC_50_ = 0.12 ± 0.007 μΜ, followed by compound **10** and **12** with IC_50_ values of 0.17 ± 0.011 and 0.24 ± 0.015 μΜ, respectively. These findings indicate that the 4-sulfonamide moiety is more favorable than the carbethoxy group for VEGFR-2 inhibition, and that chloro substitution on the 2-oxoindoline scaffold enhances activity compared to the methoxy group. Compound **9** had the most potent inhibitory effect against hCA IX and VEGFR-2. Anti-proliferative activity of compounds **9**–**12** was assessed in vitro in four human cancer cell lines derived from breast cancer (MDA-MB 231 and MCF-7), lung cancer (A549) and colorectal adenocarcinoma (Caco-2) using the MTT assay. Normal human diploid lung fibroblasts (WI-38) were used to examine compounds’ cytotoxic effect. 5-Fluorouracil (5-FU) was used as a reference drug. Results demonstrated that the tested compounds effectively inhibited the proliferation of cancer cell lines. Among the tested cell lines, MDA-MB 231 and Caco-2 exhibited the highest sensitivity with compounds **9**–**12** having IC_50_ values of 5.83 ± 0.25, 11.38 ± 0.49, 1.30 ± 0.06 and 19.38 ± 0.83 μΜ respectively against MDA-MB 231 and 18.74 ± 0.52, 4.56 ± 0.13, 2.07 ± 0.06 and 14.40 ± 0.40 μΜ respectively against Caco-2. Compound **11** was the most effective in inhibiting proliferation of MDA-MB 231 and Caco-2 cell lines. 5-FU had IC_50_ values of 18.93 ± 0.86 μΜ against MDA-MB 231 and 3.19 ± 0.09 μΜ against Caco-2. Compounds **9**–**12** showed IC_50_ values of 22.81 ± 0.71, 7.45 ± 0.23, 2.28 ± 0.07 and 20.69 ± 0.64 μΜ respectively against MCF-7 and 39.47 ± 1.14, 12.49 ± 0.36, 9.53 ± 0.28 and 36.96 ± 1.07 μΜ respectively against A549. 5-FU had IC_50_ values of 18.09 ± 0.56 μΜ against MCF-7 and 47.89 ± 1.38 μΜ against A549 (Table 8). Once again, compound **11** was the most effective in inhibiting proliferation of MCF-7 and A549 cell lines. This indicates that combination of an electron-donating group (OCH_3_) at position-5 of the 2-oxoin doline scaffold and a sulfonamide group at position-4 of the phenyl ring enhanced the anti-proliferative effect. Sensitivity order was MDA-MB 231, Caco-2 > MCF-7 > A549. Considering the safety profile of compounds **9**–**12** on WI-38 cells, they showed IC_50_ values of 80.30 ± 2.59, 34.28 ± 1.11, 31.46 ± 1.01, 41.70 ± 1.34 μΜ respectively, compared to 57.09 ± 1.84 μM for 5-FU. SI values were calculated for compounds **9**–**12**. All tested compounds, except compound **12**, exhibited low toxicity to the normal cells, with compound **11** exhibiting the highest SI values (24.20 for MDA-MB 231, 13.79 for MCF-7, 3.30 for A549 and 15.19 for Caco-2). Compound **11** inhibited Caco-2 cell migration in a wound-healing assay, showing 56.29% wound closure after 72 h. Its effect was comparable to 5-FU (62.96%) and markedly lower than the control (96.29%), indicating significant cell migration inhibition consistent with VEGFR-2 inhibition. Cytotoxic activity of compound **11** was tested in hypoxia-induced Caco-2 and MDA-MB 231 cell lines, using MTT assay. Compound **11** demonstrated potent cytotoxicity in hypoxia-induced Caco-2 with IC_50_ = 3.31 μM and MDA-MB 231 cells with IC_50_ = 5.96 μM, compared to 5-FU (IC_50_ = 15.36 μM and 19.45 μM, respectively). Flow cytometry was used to evaluate the impact of compound **11** on cell cycle distribution in Caco-2 cells. Results indicate that compound **11** arrest cell cycle at the G1 phase in a manner comparable to that of 5-FU. Annexin V-FITC/propidium iodide (AV/PI) binding capacities assay was conducted in Caco-2 cells to examine the apoptotic effect of compound **11**. Compound **11** increased the ratio of annexin V-FITC positive early apoptotic cells from 0.54% to 20.26%, while the percentage of late apoptotic cells increased from 0.22% to 11.32%. The expression level of the initiator caspase-9 was evaluated in Caco-2 cells, after treating the cells with compound **11**. Compound **11** caused a 9.92-fold increase in caspase-9 levels (from 2.58 to 25.61 ng/mL) compared to the control, exceeding the 6.58-fold increase observed with 5-FU. Furthermore, the expression levels of Bax and Bcl-2 proteins were also investigated. Compound **11** markedly increased Bax levels (6.20-fold) compared to the control, exceeding the effect of 5-FU, while significantly reducing Bcl-2 expression (4.88 vs. 16.03 in control), with a stronger effect than 5-FU. Molecular docking studies were conducted against CA IX active site of compound **11** to predict the best binding pose. Results showed scoring function = −8.97 kcal/mol of compound **9**. Molecular studies of compound **11** against VEGFR-2 revealed a binding score of −8.17 kcal/mol. Compound **11** was identified as the most active analogue, with a para-methoxy substitution on the isatin ring enhancing inhibitory activity. In addition, a para-positioned sulfonamide group further contributed to improved dual inhibition, supporting enhanced CA IX and VEGFR-2 targeting. Compound **11** exhibited inhibition of CA IX and VEGFR-2 that was slightly comparable to that of the reference drugs, indicating similar inhibitory activity toward both targets. These findings highlight compound **11** as a promising multi-target anticancer lead candidate.

### 2.4. 4-Thiazolidinones/2,4-Thiazolidinediones Carrying 2-Methylbenzenesulfonamide Derivatives

**Figure 6:**
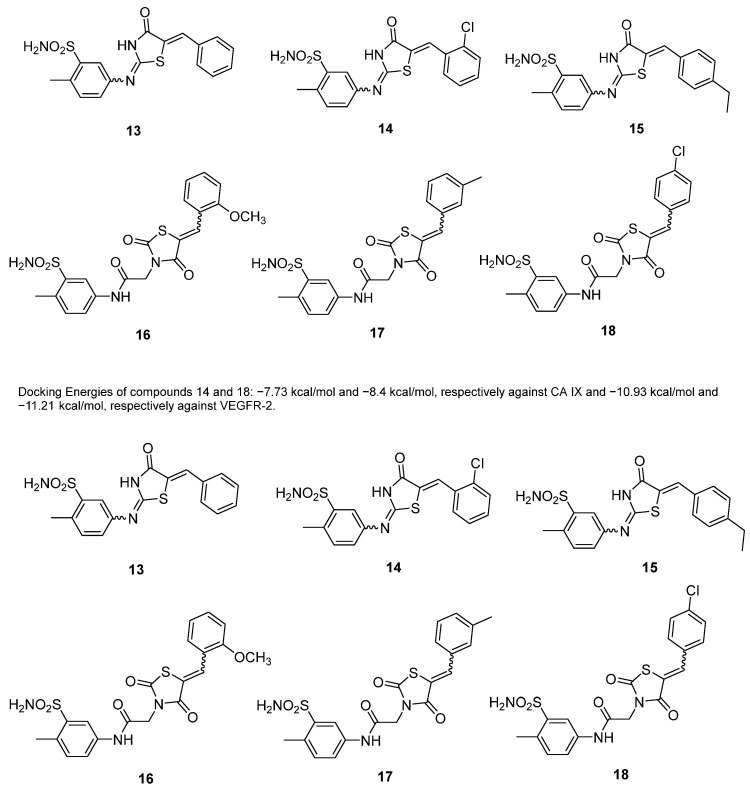
Chemical structures of 4-thiazolidinones/2,4-thiazolidinediones carrying 2-methylbenzenesulfonamide derivatives (**13**–**18**). Docking energies of compounds **14** and **18**: −7.73 kcal/mol and −8.4 kcal/mol, respectively against CA IX and −10.93 kcal/mol and −11.21 kcal/mol, respectively against VEGFR-2 [55].

In their recent research, Zengin, M. et al. (2024) [55] synthesized two series of 4-thiazolidinones/2,4-thiazolidinediones carrying 2-methylbenzenesulfonamide derivatives. The sulfonamide group was chosen as a zinc-binding motif due to its ability to coordinate with the Zn^2+^ ion in the CA active site and its reported relevance in VEGFR-2 inhibition. Five-membered heterocycles, particularly 4-thiazolidinones and 2,4-thiazolidinediones, were selected as privileged anticancer scaffolds. Compounds **13**–**18** (Figure 6) were evaluated for their CAIX inhibitory activities compared to dorzolamide (IC_50_ = 0.025 ± 0.001 µM) and AAZ (IC_50_ = 0.042 ± 0.002 µM). 4-thiazolidinones derivatives **13**–**15** showed excellent inhibitory effect with IC_50_ values of 0.056 ± 0.003, 0.035 ± 0.001, 0.073 ± 0.004 µM, respectively. Compound **14** was the most potent (IC_50_ = 0.035 ± 0.001 μM), featuring a 2-Cl substitution and showing better inhibitory activity than AAZ. 2,4-thiazolidinediones derivatives **16**–**18** revealed inhibitory activity with IC_50_ values of 0.059 ± 0.002, 0.069 ± 0.003 and 0.041 ± 0.002 µM, respectively. Compound **18**, substituted with 4-Cl, showed the best CAIX inhibitory activity comparable to that of AAZ (IC_50_ = 0.042 ± 0.002 μM). In both series, chloro substitution appears to enhance the inhibitory activity against CA IX. Compounds **13**–**18** were tested for their inhibitory effect against VEGFR-2. Sorafenib was the reference compound. Among the tested compounds, compound **18** had the best results with IC_50_ value of 0.048 ± 0.002 µM, compared to sorafenib (IC_50_ = 0.065 ± 0.003 µM). Additionally, compounds **13** and **14** exhibited potent inhibitory activity with low IC_50_ values of 0.095 ± 0.005 and 0.093 ± 0.005 μM, respectively. The cytotoxic efficacy of compounds **13**–**18** against MCF-7 cells was evaluated using the metabolic MTT assay. According to the results, compounds **14** and **18** had the most potent results with IC_50_ values of 21.32 ± 2.77 and 22.33 ± 2.09 µM, respectively, compared to sorafenib (IC_50_ = 8.39 ± 0.44 µM) and staurosporine (IC_50_ = 11.2 ± 0.51 µM). Compounds **13** and **15**–**17** exhibited promising results with IC_50_ values of 46.38 ± 3.43, 46.23 ± 2.93, 30.86 ± 3.14 and 30.51 ± 2.90 µM, respectively (Table 9). Molecular docking studies were carried out against CA IX of compounds **14** and **18**. Compounds **14** and **18** exhibited better scoring function values of −7.73 kcal/mol and −8.4 kcal/mol, respectively against CA IX. Molecular studies of compounds **14** and **18** against VEGFR-2 revealed binding scores of −10.93 kcal/mol and −11.21 kcal/mol. Physicochemical predictions of compounds **13**–**18** indicate that they possess suitable properties for a drug-likeness profile. Compounds **14** and **18** exhibited the most promising dual inhibitory activity, with effects comparable to the reference drugs. Within the 4-thiazolidinone series, compound **14** bearing a 2-chloro substituent showed a superior profile compared to the unsubstituted analogue, indicating that halogen substitution enhances activity. Similarly, in the 2,4-thiazolidinedione series, compound **18** with a 4-chloro substituent demonstrated the best overall results. This suggests that chloro substitution on the aromatic ring plays a key role in improving dual CA and VEGFR-2 inhibition. Notably, both series share a common 3-sulfonamide and 4-methyl substitution pattern, which further supports their contribution to binding affinity and overall inhibitory potency. These results can aid in the design of effective anticancer dual inhibitors targeting both CAIX and VEGFR-2.

### 2.5. Coumarin-Based Thiazole Derivatives

**Figure 7:**
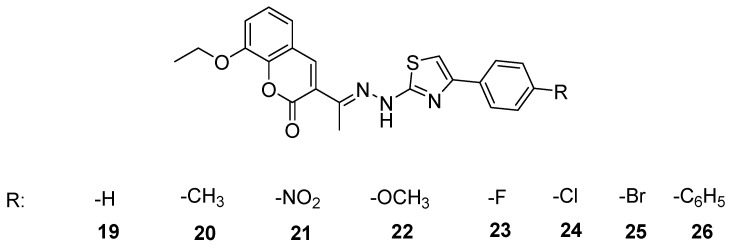
Chemical structures of coumarin-based thiazole derivatives (**19**–**26**) [56].

Hefny, S.M. et al. (2024) [56] conducted a novel class of coumarin-based thiazole derivatives. The compounds were designed to mimic sorafenib for VEGFR-2 inhibition. The coumarin scaffold was used to represent the aromatic region and linker, while the carbamimidothioate group mimic the urea interactions. Also, use of hydrophobic groups at the thiazole 4-position were picked to resemble sorafenib’s lipophilic tail and improve binding to VEGFR-2. CA (I, II, IX, and XII) inhibitory assay was assessed using a stopped-flow CO_2_ hydrase assay. AAZ and sorafenib were the reference compounds. Compounds **19**–**26** (Figure 7) were evaluated for in vitro inhibition of cytosolic hCA I and generally showed low K_I_ values, except for compound **26** (K_I_ = 78.8 μM), compared with AAZ (K_I_ = 0.25 μM) and sorafenib (K_I_ > 10 μM). Compound **26** includes biphenyl moiety. Evaluation of the cytosolic hCA II inhibition findings revealed that compounds **19**–**26** possessed weak inhibitory effect, ranging from K_I_ = 63.4 to >100 μM. Compound **20**, featuring a 4-methylphenyl group, was the most potent of the group with K_I_ value of 63.4 μM, compared with AAZ (K_I_ = 0.012 μM) and sorafenib (K_I_ ≥ 10 μM). Compounds **19**, (with unsubstituted phenyl group), **22** (with 4-methoxyphenyl group) and **23** (with 4-fluorophenyl group) were the most promising hCA IX inhibitors with excellent K_I_ values of 3.5, 3.7 and 3.1 μM, respectively. Compounds **20**, **21**, **24**–**26** showed good K_I_ values of 4.5, 6.7, 6.1, 8.7 and 6.9 μM, respectively compared with AAZ (K_I_ = 0.025 μM) and sorafenib (K_I_ = 8.1 μM). The introduction of a second phenyl moiety at the para position (compound **26**) decreased inhibitory activity toward isoform IX compared to the unsubstituted phenyl analogue (compound **19**). Also, the inhibitory activity toward IX is anticipated in accordance with the atom’s radius. This explains the higher inhibitory activity of compound **23** (K_I_ = 3.1 μM), which contains a fluorine atom (smallest radius), compared to compound **24** bearing chlorine (K_I_ = 6.1 μM) and compound **25** bearing bromine (K_I_ = 8.7 μM), which shows the lowest activity. Attaching electron-donating groups, such as the methoxy group (compound **22**, K_I_ = 3.7 μM) and the methyl group (compound **20**, K_I_ = 4.5 μM) resulted in better activity than the electron-withdrawing group nitro group (compound **21**, K_I_ = 6.7 μM). Compounds **19**–**26** showed great inhibitory activity against the isoform hCA XII with K_I_ values of 2.8, 3.7, 6.5, 5.2, 1.5, 3.8, 4 and 9.1, respectively compared with AAZ (K_I_ = 0.005 μM) and sorafenib (K_I_ ≥ 10 μM). The 4-fluorophenyl (**23**) analogue showed stronger inhibitory effect (K_I_ = 1.5 μM) among the tested compounds. Incorporation of extra phenyl moiety to the phenyl ring at compound **19** (K_I_ = 2.8 μM), as in biphenyl analogue (**26**, K_I_ = 9.1 μM), reduced the inhibitory effect. Increasing the halogen atomic radius in halogenated phenyl derivatives (**23**–**25**) resulted in reduced inhibitory activity, with potency decreasing from fluorine (**23**, K_I_ = 1.5 μM) to chlorine (**24**, K_I_ = 3.8 μM) and bromine (**25**, K_I_ = 4.0 μM). Attaching electron-donating groups, such as the methyl group (compound **20**, K_I_ = 3.7 μM) and the methoxy group (compound **22**, K_I_ = 5.2 μM) resulted in better activity than the electron-withdrawing group nitro group (compound **21**, K_I_ = 6.5 μM) (Table 10). SI rations were calculated for compounds **19**–**26**. The SRI/IX of compounds **19**–**26** ranged from 11.4 to 33.3. All the compounds showed higher selectivity when compared with AAZ (SRI/IX = 10). The phenyl-substituted compound **19** showed high selectivity toward hCA IX (SRI/IX = 28.5), nearly threefold higher than AAZ. Introducing a small electron-withdrawing group such as fluorine (compound **23**) further improved selectivity (SRI/IX = 33.3). In contrast, larger electron-withdrawing groups, such as chloro (compound **24**) and bromo (compound **25**), reduced selectivity to 16.3 and 11.4, respectively. Similarly, the nitro-substituted compound **21** showed decreased selectivity (SRI/IX = 14.9) compared to the unsubstituted analogue **19**. Among electron-donating groups, the methoxy derivative (compound **22**) displayed better selectivity than the methyl analogue (compound **20**), with SRI/IX values of 27.0 and 22.2, respectively. However, adding an additional phenyl ring, as in the biphenyl analogue **26**, led to a marked reduction in selectivity (SRI/IX = 11.4) compared to compound **19**. The computed SRI/XII for compounds **19**–**26** ranged from 8.6 to 66.6. Compound **23** with a 4-fluoro group possessed the highest hCA XII selectivity, with SRI/XII = 66.6 (compared with AAZ, SRI/XII = 43.8). Larger halogens acting as electron-withdrawing groups, such as chloro (compound **24**) and bromo (compound **25**), led to a noticeable decrease in selectivity (SRI/XII = 26.3 and 25.0 respectively) compared to the smaller fluoro analogue (compound **23**, SRI/XII = 66.6). The unsubstituted compound **19** showed the second highest selectivity (SRI/XII = 35.7). Regarding substituent effects at the para position of the phenyl moiety, electron-donating groups, such as methyl (compound **20**, SRI/XII = 27.0) and methoxy (compound **22**, SRI/XII = 19.2), provided higher selectivity than the electron-withdrawing nitro group (compound **21**, SRI/XII = 15.3). In contrast, introducing an additional phenyl ring, as in the biphenyl analogue **26**, significantly reduced selectivity (SRI/XII = 8.6) compared to compound **19**. The synthesized compounds revealed high selectivity against hCA IX over II, with SRII/IX from 9 to 27.0 relative to AAZ, SRII/IX = 0.5. All analogues showed great selectivity with SRII/XII from 10.9 to 49.5 relative to AAZ (SRII/XII = 2.1) (Table 11). Compounds **19**, **22** and **23** were selected to be assessed for their inhibition action on VEGFR-2 in vitro, with sorafenib (IC_50_ = 30.0 ± 0.09 nM) being the reference compound. Compounds **19**, **22** and **23** showed strong inhibitory effect against VEGFR-2, with compound **23** being the most potent with IC_50_ of 32.1 ± 1.10 nM. Surprisingly, adding methoxy moiety (compound **22**) markedly enhanced the inhibitory potency to an IC_50_ value equal to 34.8 ± 1.04 nM compared to the unsubstituted phenyl derivative (compound **19**), which had an IC_50_ of 68.6 ± 1.80 nM. Under hypoxic conditions, compounds **19**, **22** and **23** were further evaluated for their possible in vitro antiproliferative effect against PANC1 (pancreatic cancer), MCF7 (breast cancer), and PC3 (prostate cancer) cell lines. DOX was the drug control, using the MTT assay. The results showed that compounds **19** and compound **23** exhibited similar or slightly improved growth inhibitory activity against PANC-1 cells, with IC_50_ values of 1.07 and 1.04 μM, respectively. Against MCF-7 cells, the IC_50_ values were 1.66 and 1.40 μM, respectively, remaining comparable to the reference drug DOX (IC_50_ = 6.90 μM for PANC-1 and 4.17 μM for MCF-7). In contrast, introducing a methoxy group at the para position, as in compound **22**, reduced antiproliferative activity, with IC_50_ values of approximately 1.82 μM (PANC-1) and 3.01 μM (MCF-7). Overall, the tested compounds showed lower inhibitory effects against PC-3 cells, with IC_50_ values above 5 μM, compared to DOX (IC_50_ = 8.87 μM). Also, compound **23** significantly inhibited PANC-1 cell migration in wound healing and transwell assays compared to the control, suggesting its potential to limit metastasis. Furthermore, compound **23** urged apoptosis of pancreatic cancer (PANC1) by increasing proapoptotic factors (caspase 3/8/9, p53, Cytochrome C, and BAX) and suppressing the antiapoptotic factors (Bcl-2) and induced the cell cycle arrest in the S phase. Anticancer effectiveness of compound **23** was conducted in vivo in female mice with SEC tumors. Treatment of compound **23** improved biochemical, hematological, histological, and immunohistochemical parameters, and significantly decreased the weight and volume of the tumors. Compound **23** had been docked within VEGFR-2 and CA IX receptors, to predict the best binding mode, with scoring functions of −153.609 ± 20.117 kJ/mol and −128.247 ± 13.682 kJ/mol, respectively. The ADMET features of compound **23** were predicted using ADMETlab 2.0, showing outstanding ADMET properties. Compound **23** exhibited the most potent dual inhibitory activity, with IC_50_ values lower than those of the reference drugs. The presence of a fluoro group at the 4-position was associated with superior activity compared to the corresponding chloro and bromo analogues. This may be attributed to the small size and strong electron-withdrawing effect, which can enhance binding interactions of both CA IX and VEGFR-2.

### 2.6. Sulfonamide-Triazole-Glycoside Hybrid Derivatives

**Figure 8:**
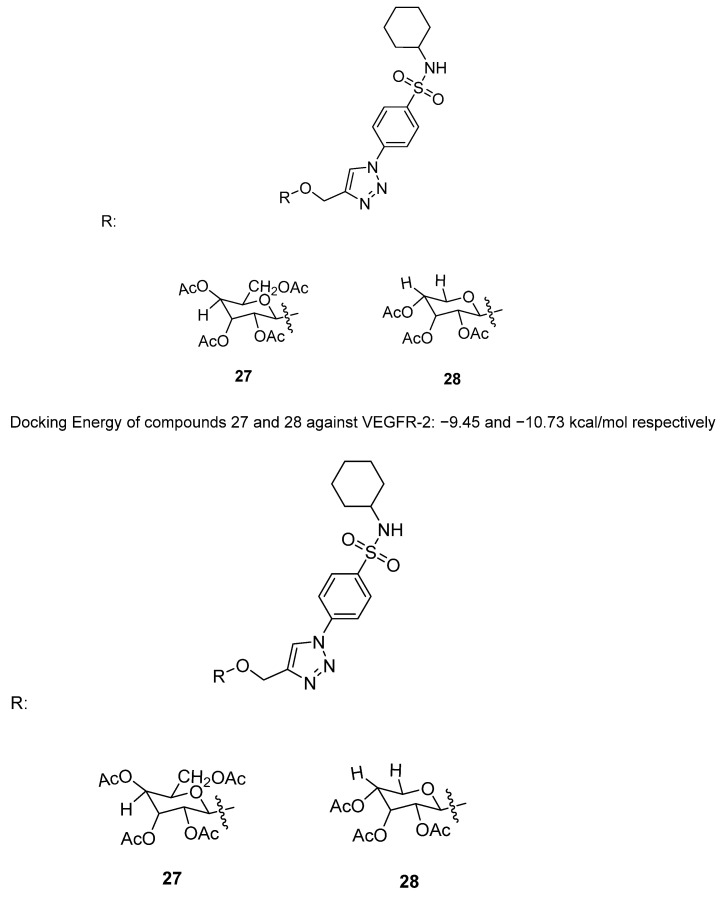
Chemical structures of sulfonamide-triazole-glycoside hybrid derivatives (**27**, **28**). Docking energy of compounds **27** and **28** against VEGFR-2: −9.45 and −10.73 kcal/mol respectively [57].

Abbas, H.A.S. et al. (2024) [57] developed and analyzed a series of new sulfonamide-triazole-glycoside hybrid derivatives. Sulfonamides and 1,2,3 triazoles were selected due to their wide range of biological activities. Also, the addition of glycosides to heterocyclic compounds has generated significant hybrids with biologically valuable characteristics, particularly antiviral, anticancer, and antibacterial properties. Compounds **27** and **28** (Figure 8) were evaluated for their in vitro inhibitory potency against VEGFR-2 and the CA isoforms hCA IX and hCA XII. Compound **27** and **28** showed promising inhibitory effects against hCA IX with IC_50_ values of 66 and 40 nM respectively compared to SLC-0111 (IC_50_ = 53 nM). Compounds also exhibited excellent results against hCA XII with IC_50_ values of 7.6 and 3.2 nM respectively compared to SLC-0111 (IC_50_ = 4.8 nM). In vitro inhibitory assessment of compounds **27** and **28** against VEGFR-2 showed excellent results with IC_50_ values of 1.33 ± 0.10 and 0.38 ± 0.14 µM respectively. Sorafenib was used as the reference compound with IC_50_ = 0.43 ± 0.10 µM. Lung A-549, liver HepG-2, breast MCF-7 and colorectal HCT-116 cancer cell lines were used to test preliminary cytotoxic efficacy of compounds **27** and **28**, in vitro through MTT assay. DOX and sunitinib were used as standard drugs. Compounds **27** and **28** demonstrated promising activity against HepG-2 and MCF-7 (IC_50_ = 10.45 ± 0.13 and 8.39 ± 0.20 µM, respectively, against HepG-2 and 20.31 ± 0.66 and 21.15 ± 2.45 µM, respectively, against MCF-7), comparing with DOX (IC_50_ = 13.76 ± 0.45 and 17.44 ± 0.46 µM against HepG-2 and MCF-7, respectively) (Table 12). The ability of compound **28** to induce apoptosis was evaluated by treating MCF-7 cells for 24 h at a concentration of 21.15 μM. The cells were also examined through flow cytometry with annexin-V staining. Results indicated that compound **28** was able to arrest MCF-7 cells in the G2/M phase of the cell cycle. Compound **28** markedly increased both early and late apoptosis in MCF-7 cells compared to the DMSO control, along with a slight rise in necrosis. Overall, the data indicate that compound **28** induces cell death mainly through apoptosis. MCF-7 cells were treated for 24 h with compound **28** exhibited a 6.2-fold increase in Bax levels (271.45 Pg/mL) compared to untreated control cells (43.66 Pg/mL). Also, MCF-7 cells were treated with compound **28** and it was observed that Bcl-2 protein was downregulated by 2.6 times, going from 8.51 to 3.27 ng/mL. Additionally, compound **28** enhanced the p53 protein level by 7.4 times in MCF-7 treated cells compared to 125.40 Pg/mL in control cells. Among the synthesized compounds, cyclohexane substitution on the sulfonamide moiety showed the most favorable effects. In contrast, within the glycoside group, the presence of an acetoxymethyl group at the 2-position along with the acetate configuration at the 3-position reduced cytotoxic activity as well as hCA IX, hCA XII, and VEGFR-2 inhibition. Compounds **27** and **28** displayed the best binding mode inside the VEGFR-2 active site with a docking score of −9.45 and −10.73 kcal/mol, respectively. Regarding CA isoforms hCA IX and hCA XII, the sulfonamide group in compounds **27** and **28** strengthened binding through ionic interaction with the zinc ion. Considering the glycoside group, compound **28**, with hydrogen substitution at C2 and C3, showed slightly improved dual IC_50_ values compared to the other analogues and even better activity than the reference drugs.

### 2.7. 1,5-Diaryl-1,2,4-Triazole-Tethered Sulfonamides Derivatives

**Figure 9:**
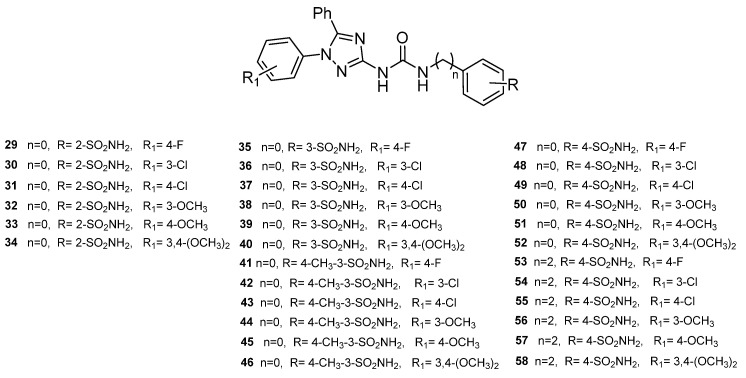
Chemical structures of 1,5-diaryl-1,2,4-triazole-tethered sulfonamides derivatives (**29**–**58**) [58].

New 1,5-diaryl-1,2,4-triazole-tethered sulfonamides derivatives were synthesized and assessed by Elsawi, A.E. et al. (2023) [58]. The derivatives were designed using a “tail approach” to improve CA IX inhibition. The sulfonamide group was used as the zinc-binding part for strong interaction with the enzyme. The triazole and aryl groups were conducted as flexible tails to better fit the active site and increase potency and selectivity toward hCA IX. Compounds were evaluated for their potential inhibitory activity against a panel of hCAs (hCA I, II, IX, and XII isoforms) applying stopped-flow CO_2_ hydrase assay. AAZ was used as a reference compound. Considering the inhibitory effect against the hCA I, it was observed that compounds **29**–**58** (Figure 9) displayed different inhibitory activities ranging from 39.8 nM to >10 μM. All the tested compounds **29**–**34**, bearing ortho sulfamoyl functionality or *meta* sulfamoyl parallel with *para* tolyl moiety compounds **41**–**46** did not demonstrate potent hCA I inhibitory activity up to K_I_ of 10 μM relative to 250 nM for AAZ. Compounds **35**–**40** with *meta* sulfamoyl substitution possessed weak inhibitory activities ranging from 4.18 μM to >10 μM. Change of sulfamoyl moiety from *ortho* position compounds **29**–**34** (K_I_ values > 10 μM) to the *meta* position, compounds **35**–**40** enhanced the inhibitory effect (K_I_ range 4.18–9.42 μM, expect form compound **40**. Similarly, compounds **47**–**52** attached to the *para*-sulfamoyl moiety showed weak inhibitory activity, with K_I_ values ranging from 1.94 μM for the *para*-chloro compound **49** to 5.4 μM for the 3,4-dimethoxy compound **52**. Introduction of a *para*-sulfamoyl group had a stronger effect on inhibitory activity compared to the *ortho* (compounds **29**–**34**) and meta (compounds **35**–**40**) derivatives. Compounds **53**–**55** and **57** showed potent hCA I inhibition, with K_I_ values of 39.8, 61.8, 81.4, and 82.7 nM, respectively, whereas compound **56** and compound **58** displayed moderate activity (K_I_ = 100.7 and 152.7 nM, respectively). Inhibitory effect of compounds **29**–**58** against hCA II displayed potent results with inhibitory K_I_ values ranging from 0.9 to 547.2 nM compared to AAZ (K_I_ = 12 nM). For hCA II, *para*-sulfamoyl compounds **47**–**52** were more effective than both *ortho* and *meta* analogues, with activity influenced by phenyl substitution, where methoxy-substituted compounds performed best. Compound **57**, featuring a *para*-methoxy group, showed the strongest hCA II inhibition overall with K_I_ value of 0.9 nM. The in vitro inhibition of compounds **29**–**35** against hCA IX showed K_I_ values ranging from 54 to 179 nM, compared with the standard AAZ (K_I_ = 25 nM). Among these, the 3-methoxy-substituted N-phenyl compound **32** showed the most favorable inhibition, followed by the 4-fluoro (compound **29**), 4-methoxy (compound **33**), 4-chloro (compound **31**), 3-chloro (compound **30**), and 3,4-dimethoxy (compound **34**) analogues, with K_I_ values of 54, 75.9, 92, 100.2, 119.8, and 179.7 nM, respectively. Shifting the sulfamoyl group from the *ortho* (compounds **29**–**34**) to the *meta* position (compounds **35**–**40**) generally improved inhibitory activity. The most potent compounds were the 4-fluoro and 4-methoxy derivatives (compound **35** and **38**) with K_I_ values of 32.1 and 38.4 nM, respectively, while the remaining analogues (compounds **36**–**37** and **39**–**40**) also showed good inhibition with K_I_ values ranging from 48.6 to 69.7 nM. Adding a *para*-methyl group to the *meta*-benzenesulfonamide in compound **35** (K_I_ = 32.1 nM) led to a slight improvement in activity, as seen in compound **41** (K_I_ = 28 nM). However, applying the same modification to the *meta*-methoxy compound **38** (K_I_ = 38.4 nM) reduced its potency to compound **44** (K_I_ = 63.7 nM). Similarly, compounds **42**, **43**, and **45**–**46** showed weaker inhibition, with K_I_ values of 67.2, 84.2, 81.7, and 125.7 nM, respectively, compared to their unsubstituted analogues. Notably, relocating the sulfamoyl group to the *para* position enhanced inhibitory activity compared to both *ortho* and *meta* compounds, with K_I_ values ranging from 5.2 to 38.1 nM. *Para*-sulfamoyl derivatives exhibited strong hCA IX inhibition, with the 4-fluoro compound **47** being the most active, while other substitutions (methoxy and chloro) led to reduced activity. Compounds **53**–**58**, bearing a linker generally enhanced activity, with the 4-fluorophenyl compound **53** emerging as the most potent (K_I_ = 2.2 nM), although it also strongly inhibited hCA II. Replacing the 4-fluoro group with chloro or methoxy substituents slightly decreased hCA IX inhibition but improved selectivity by reducing activity against hCA II. For hCA XII inhibition, the *ortho* compounds **29**–**35** showed moderate activity, while shifting the sulfamoyl group to the *meta* position (compounds **35**–**46**) generally improved potency, although the effect depended on the substituent. Introducing a *para*-sulfamoyl group (compounds **47**–**52**) led to a enhancement, giving the most active compounds with low K_I_ values, especially the 4-fluoro (compound **47**) and methoxy-substituted (compound **51**) analogues with K_I_ values of 4.7 and 5.6 nM. In compounds **53**–**58**, extending the linker improved activity for some derivatives, with the 4-methoxy compound (compound **57**) emerging as the most potent hCA XII inhibitor (K_I_ = 3.2 nM). The selectivity of compounds **29**–**58** was evaluated for the isoforms hCA IX and XII relative to the cytosolic isoforms hCA I and II. Overall, the compounds showed moderate to excellent selectivity toward hCA IX and XII over hCA I, with SI values ranging from 4.8 to 982.3 and 3.2 to 740.3, respectively. They submitted the compounds to the NCI Developmental Therapeutic Program for screening of growth-inhibitory potential against several cancer cell panels to further investigate their in vitro anti-proliferative activities. Antiproliferative activity was evaluated following the guidelines of the NCI Bethesda Drug Evaluation Branch, using a panel of 60 cancer cell lines to screen all compounds at 10 µM. The most potent compounds **31**, **33**, **42**, **43**, **45** and **47**–**49** were used to investigate their potential VEGFR-2 inhibition utilizing a colorimetric enzyme-linked immunosorbent assay (ELISA). Sunitinib was used as a reference drug. Compounds **42** and **47** were the most potent with IC_50_ values of 96.2 ± 2 and 26.3 ± 0.4 nM, respectively compared with sunitinib (IC_50_ = 39.7 ± 2 nM). Additionally, compounds **31**, **33** and **49** displayed a good VEGFR-2 inhibitory activity with IC_50_ values of 271 ± 14.0, 183 ± 8.1, 318.0 ± 13 nM, respectively. Compounds **43** and **45** exhibited moderate inhibitory effect (IC_50_ values = 541 ± 23.0 and 717 ± 30.0 nM, respectively). Finally, compound **48** displayed the weakest VEGFR-2 inhibitory activity among the tested compounds (IC_50_ > 1000 nM). Compounds **31**, **33**, **42**, **43**, **45** and **47**–**49** were tested in vitro for anti-proliferative action against breast MCF-7 and T47D cancer cell lines, using the MTT assay. Staurosporine was used as a reference compound. Compounds **31**, **33**, **47**, and **49** showed strong antiproliferative activity against MCF-7 and T47D cell lines. Against MCF-7, the IC_50_ values were 2.19 ± 0.09, 1.06 ± 0.04, 0.66 ± 0.04, and 2.17 ± 0.09 μM, respectively, while against T47D they were 2.97 ± 0.12, 2.34 ± 0.09, 4.51 ± 0.2, and 2.84 ± 0.11 μM, respectively. Compound **47** exhibited potent inhibitory effects on hCA IX and VEGFR-2 (KI = 4.7 nM and IC_50_ = 26.3 nM, respectively), also exhibited excellent antiproliferative activity against MCF-7 cell lines (IC_50_ = 0.66 ± 0.04 μM). Also, compounds **42**, **43**, **45** and **48** demonstrated good activity against MCF-7 cell lines (IC_50_ values = 10.9 ± 0.62, 7.72 ± 0.32, 6.86 ± 0.28, and 19.0 ± 1.08 μM, respectively) and T47D (IC_50_ values = 17.9 ± 0.78, 11.6 ± 0.47, 18.7 ± 0.75, and 23.9 ± 1.04 μM, respectively). A cell growth inhibitory assay was conducted on the non-tumorigenic human breast epithelial cell line (MCF-10A), to assess the selectivity of compounds **31**, **33**, **42**, **43**, **45** and **47**–**49**. All the compounds showed selectivity toward breast cancer MCF-7 cells when compared to MCF-10A cells. Compounds **33**, **47**, and **49** exhibited remarkable selectivity, with high SI values of 44.81, 38.75, and 29.93, respectively (Table 13). Compounds **31**, **42**, **43**, **45**, and **48** possessed good selectivity, with SI value of 14.47, 7.08, 5.08, 9.04 and 4.37, respectively. Molecular docking studies were conducted on compound **47** in the three enzymes: hCA IX, hCA XII, and VEGFR-2. Results revealed small RMSD values of 0.504, 0.98, and 0.20 Å between the docked poses and the co-crystallized ligands of hCA IX, XII, and VEGFR-2, respectively, indicating an almost identical alignment, confirming the reliability of the applied setup for the planned docking studies. Compound **47** showed the best dual inhibitory profile, successfully inhibiting both VEGFR-2 and CA IX with IC_50_ values lower than those of the reference drugs. The compound bears a 4-sulfonamide moiety and a para-fluoro substituent, suggesting that the smaller size of fluorine contributes favorably to activity. Also, compound **47** showed good selectivity toward hCAIX and XII isoforms over the off target hCAI isoform. Molecular docking analysis studied the binding of compound **47** inside hCAIX and XII and VEGFR-2 active sites and supported the acquired CA and VEGFR-2 kinase inhibitory actions.

### 2.8. Indolinone-Based Benzenesulfonamide Derivatives

**Figure 10:**
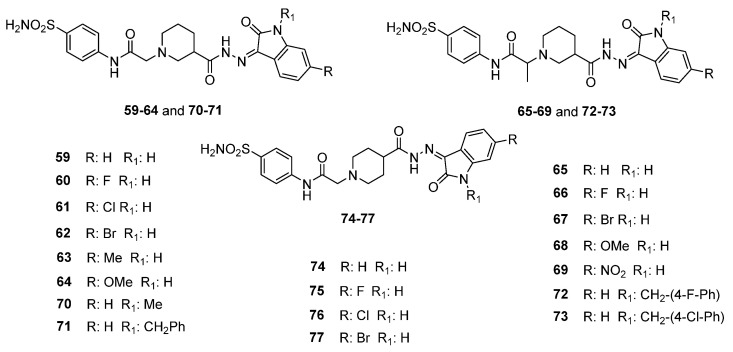
Chemical structures of indolinone-based benzenesulfonamide derivatives (**59**–**77**) [59].

Saied, S. et al. (2023) [59] constructed a novel class of novel indolinone-based benzenesulfonamide derivatives. The isatin scaffold was chosen because it is a well-known anticancer structure that can inhibit both hCA IX/XII and VEGFR-2. It is also present in approved drugs like nintedanib and sunitinib, which target VEGFR-2-related pathways. Previous isatin–sulfonamide compounds have shown strong activity against hCA IX/XII, and indolinone-based benzenesulfonamides were designed to combine isatin anticancer activity with sulfonamide zinc-binding ability for dual inhibition. Compounds **59**–**77** (Figure 10) were assessed for their inhibitory potential toward hCA I and hCA II isoforms, in addition to hCA IX and XII, using stopped-flow CO_2_ hydrase assay. AZZ was the reference compound. Compounds **59**–**77** displayed a wide range of inhibitory potential with K_I_ value range starting from 25.1 nM to 369.8 nM compared to AAZ (K_I_ = 250 nM). Regarding the *meta* derivatives **59**–**64**, they displayed inhibitory activities with K_I_ values ranging from 62.3 to 136.9 nM. Compound **60**, bearing a 5-fluoro moiety, was the most potent (K_I_ = 62.3 nM), and compound 60, with 5-bromo moiety, was the worst (K_I_ = 136.9 nM). N-methylation slightly enhanced the inhibitory effect (compound **70**, K_I_ = 71.2 nM) relative to the unsubstituted derivative **59** (K_I_ = 82.1 nM). Shifting of *meta* acetamide linker in compounds **59**–**64** (K_I_s from 62.3 to 136.9 nM) to *para* compounds **74**–**77** moderately enhanced the activity (K_I_s from 35.5 to 132.8 nM) with compound **75**, having 5-fluoro moiety, being the best one (K_I_ = 35.5 nM). Compound **68**, with propanamide linker and 5-methoxy group, was the most potent of all the synthesized compounds with K_I_ value of 25.1 nM). Most of the synthesized compounds showed strong inhibition of hCA II. The *meta*-analogues **59**–**64**, which displayed good inhibitory effect with KIs values spanning from 0.75 to 58.2 nM. Structural modifications such as isatin N-substitution and linker changes had mixed effects on activity, sometimes improving and sometimes reducing potency. *Para* acetamide derivatives **74**–**77** generally showed comparable or slightly improved activity over their *meta* counterparts, with the 5-chloro compound **76** exhibiting the strongest inhibition (K_I_ = 0.8 nM). Overall, the presence of a 5-chloro substituent appears to be a key factor for enhanced hCA II inhibitory activity, as shown by compounds **61** and **76** (K_I_ = 0.75 and 0.8 nM, respectively), compared with AAZ (K_I_ = 12 nM). Compounds **59**–**77** were tested for their inhibitory activity against hCA IX and presented K_I_ values ranging from 3.6 to 65.9 nM, compared with AZZ with K_I_ = 25 nM. Among the *meta* acetamide compounds **59**–**64**, adding a methyl group at position 5 of the isatin ring produced the most potent hCA IX inhibitor in this series (K_I_ = 15.8 nM), followed by the 5-methoxy, 5-chloro, and 5-fluoro analogues, with K_I_ values of 19, 22.1, and 35.4 nM, respectively. Compounds **59**, with unsubstituted isatin and compound **62**, with 5-bromo substituent, were the least active compounds of this series (K_I_s = 52.8 and 65.9 nM, respectively). Methyl branching of the flexible linker to provide the *meta* propanamides **65**–**69** significantly enhanced the hCA IX inhibitory activity. Compound **65**, with unsubstituted isatin, was the most potent hCA IX inhibitor of all the synthesized compounds with K_I_ value of 3.6 nM. Compound **65** was followed by compound **68**, with 5-methoxy substitution and compound **66**, with 5-fluoro substitution and compound **67**, with 5-bromo substitution (K_I_s values = 4.2, 5.1 and 9.8 nM, respectively). Shifting from *meta* acetamides **59**–**62** (KIs = 52.8, 35.4, 22.1 and 65.9 nM, respectively) to afford the *para* analogues **74**–**77** improved hCA IX inhibitory effect with K_I_s values of 24.8, 6.2, 14.3, 27.9 nM, respectively. Compound **75** was the most potent from this series with K_I_ value of 6.2 nM. All tested compounds showed good hCA XII inhibition with compound **60**, being the most potent with K_I_ = 0.93 nM. Other substitutions on the isatin ring, such as methoxy, chloro, and methyl, also showed good activity but at slightly higher K_I_ values. Modifying the linker from acetamide to branched propanamide or introducing *N*-substitution generally reduced potency. In addition, shifting from *meta* to *para* acetamides decreased activity, although some *para* derivatives, particularly the 5-chloro derivative, still showed strong inhibition. Compounds **65**, **68** and **75** were selected to be evaluated for their in vitro inhibitory effect against VEGFR-2, using colorimetric Enzyme-Linked Immunosorbent Assay (ELISA). Sorafenib was used as a refence drug. Compounds showed moderate inhibitory effect with compound **65**, non-substituted *meta* branched propanamide, having the best one with IC_50_ values of 204 ± 9 nM, compared with sorafenib (IC_50_ = 41 ± 2 nM). Also, the introduction of methoxy group to position 5 of isatin compound **68** significantly decreased the inhibitory effect with IC_50_ value of 358 ± 16 nM. Notably, introducing a 5-fluoro isatin together with a *para* acetamide linker (compound **75**) significantly reduced VEGFR-2 inhibitory activity, giving an IC_50_ value of 811 ± 36 nM. Compounds **65**, **68** and **75** were further screened for their potential in vitro anti-proliferative effect against MDA-MB-231 and MCF-7 breast cancer cell lines under hypoxic conditions. MTT assay was used using 5-FU as a positive drug control. Compound **65**, the non-substituted *meta* branched propanamide, possessed better growth inhibitory effect against MDA-MB-231 cell lines with IC_50_ value of 4.083 ± 0.175 μM compared to the standard drug (5-FU with IC_50_ = 8.704 ± 0.372 μM), while demonstrated IC_50_ value of 9.997 ± 0.364 μM against MCF-7 cell lines comparable to the standard (5-FU with IC_50_ = 5.167 ± 0.188 μM). Compound **68** retained moderate activity, showing slightly better activity than the reference compound against MDA-MB-231 (IC_50_ = 7.204 ± 0.308 μM) but weaker activity against MCF-7 (IC_50_ = 11.345 ± 0.413 μM). In contrast, introducing a 5-fluoro isatin with a *para* acetamide linker, compound **75**, significantly reduced activity against both cell lines (IC_50_ values of 18.853 ± 0.807 μM against MDA-MB-231 and 29.292 ± 1.067 μM against MCF-7) (Table 14). The apoptotic impact of compound **65** was tested on the apoptotic markers Bax, Bcl-2, and active caspase-3. The results showed that treatment with compound **65** altered the expression levels of the examined proteins (Bax, Bcl-2, and active caspase-3). Molecular docking of compounds **65**, **67**, and **75** against hCA IX and VEGFR-2 revealed key interactions, including zinc coordination via the sulfonamide group and multiple hydrogen bonding and hydrophobic contacts within the active sites. Compound **65** exhibited the best dual inhibitory activity among the three series, suggesting that the propanamide linker enhances CA and VEGFR-2 inhibition. In addition, the unsubstituted analogue showed the most favorable profile. The presence of a methyl group increases steric bulk and hydrophobicity, which may contribute positively by improving hydrophobic interactions and optimizing binding within the active site.

### 2.9. Isatin-Based Sulfonamide Derivatives

**Figure 11:**
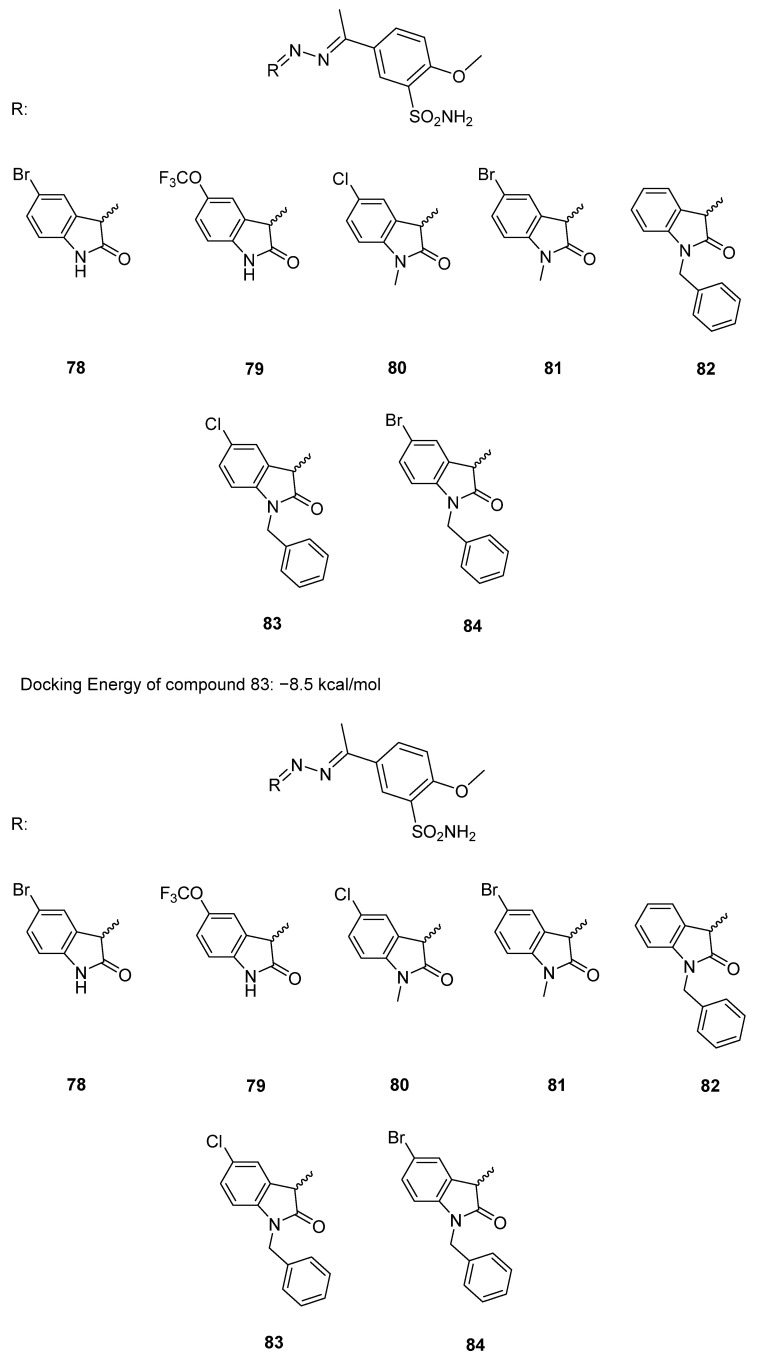
Chemical structures of isatin-based sulfonamide derivatives (**78**–**84**). Docking energy of compound **83**: −8.5 kcal/mol [60].

Shaldam, M.A. et al. (2023) [60] synthesized a new set of isatin-based sulfonamide derivatives. Isatin was selected as a privileged scaffold due to its well-documented anticancer properties and its reported ability to inhibit both hCA IX/XII and VEGFR-2. The sulfonamide moiety was incorporated as a classical zinc-binding group in the CA active site. Together, these features were combined to design dual-target inhibitors capable of simultaneously modulating CA and VEGFR-2 pathways for improved anticancer activity. Compounds **78**–**84** (Figure 11) were tested for their in vitro anti-tumor activity against the NCI panel, including 58 different human tumor cell lines representing nine types of cancer, at a single concentration of 10 µM. Apart from CNS cancer with compound **81** and leukemia with compounds **83**–**84**, all the compounds showed the highest growth inhibition against breast cancer cell lines. T47D cells exhibited the greatest sensitivity, displaying the highest GI% values in the single-dose in vitro assay for compounds **78**–**84** (**54**, **32**, **55**, **52**, **57**, **28** and **39** respectively). A dose-response analysis was conducted on T47D, breast cancer cells, using the sulforhodamine B colorimetric assay. Compounds **78**–**84** exhibited high to moderate inhibitory activity, with IC_50_ values ranging from 1.83 to 24.13 µM, respectively, with compound **80** being the most potent, with IC_50_ value of 1.83 ± 0.08 µM, compared with DOX, with IC_50_ value of 2.26 ± 0.10 µM. Considering the single-dose assay, the results indicated that the 5-Br derivative was preferred over the N-(un)alkylated derivatives, and compound **78** (IC_50_ = 5.45 ± 0.24 µM) was the most potent of the series. From the N-alkylated/arylated isatin derivatives with methyl or benzyl groups, chloro substitution at position 5 led to higher activity, with the N-methyl compound **80** demonstrating an IC_50_ value of 1.83 ± 0.08 µM and the N-benzyl compound **83** an IC_50_ value of 3.59 ± 0.16 µM. Compounds **78**, **80**, **81** and **83** were evaluated for inhibitory activity against hCA I, II, and IX, and none of the synthesized compounds showed inhibition (K_I_ > 100 µM) toward any of the isoforms. AAZ was used as the refence drug. The lack of activity was possibly due to steric hindrance caused by the adjacent methoxy group. Compounds **78**, **80**, **81** and **83** were evaluated for their in vitro VEGFR-2 inhibition activities, via stopped flow assay. Results showed IC_50_ values of 56.70 ± 0.72, 63.40 ± 0.72, 30.10 ± 0.31 and 23.10 ± 0.41 nM, respectively. Compound **83** possessed the highest inhibitory activity (IC_50_ = 23.10 ± 0.41 nM), followed by compound **81** (IC_50_ = 30.10 ± 0.31 nM) compared with sorafenib (IC_50_ = 29.70 ± 0.17 nM). T47D cells treated with compound **80** exhibited an increase in the sub-G1 and G0/G1 phases (45.88% and 68.42%, respectively) compared with the control (DMSO), which showed 61.39% and 2.41%, respectively. Compound **83** bearing a phenyl moiety attached to ethylindolin-2-one demonstrated the best VEGFR-2 inhibitory effect with IC_50_ = 23.10 ± 0.41 nM, while 5-chloro substitution of 1-methylindolin-2-one (compound **80**) reduced inhibitory activity with IC_50_ = 63.40 ± 0.72 nM compared to the 5-Br substitution (compound **81**) with IC_50_ = 30.10 ± 0.31 nM (Table 15). Molecular docking studies in the active site of the VEGFR-2, were conducted on compound **83**, to find the best binding pose, and showed a docking score of −8.5 kcal/mol. While the target isatin sulfonamides potently inhibited VEGFR-2, they failed to inhibit the CA isoforms, contrary to predictions. Spatial architecture, linker flexibility, and target pocket geometry dictate success. This could be attributable to steric hindrance from the neighbouring methoxy group adjacent to the sulfanilamide moiety, as well as the overall geometry of the compound, which compromises the precise geometric requirements needed for CA IX active-site binding.

## 3. Dual CA IX and EGFR Inhibitors

### 3.1. Sulfamoylphenyl-Dihydro-Thiadiazole Derivative

**Figure 12:**
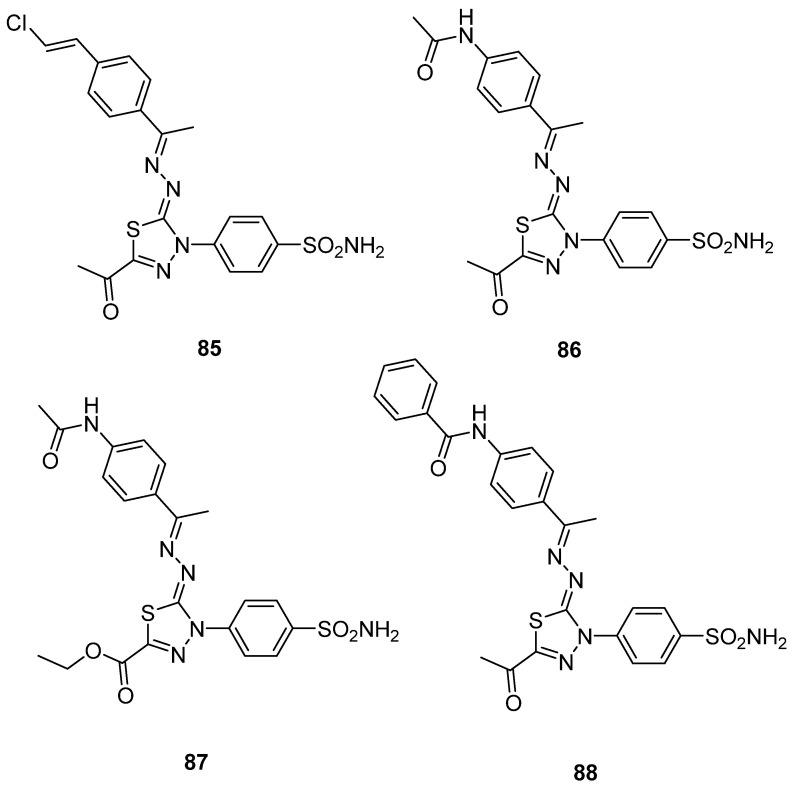
Chemical structures of sulfamoylphenyl-dihydro-thiadiazole derivative (**85**–**88**) [61,62,63,64].

Expanding on their previous research, Eissa, I.H. et al. (2026) [61], developed a novel 2,3-dihydro-1,3,4-thiadiazole-benzenesulfonamide hybrid. Biological assays demonstrated potent inhibitory activity of compound **85** (Figure 12) with IC_50_ values of 0.039 μM, 0.04 μM, and 0.05 μM for CAIX, CAXII, and EGFR, respectively, comparable or superior to standard inhibitors (AAΖ and erlotinib) (Table 16). In vitro cytotoxicity studies demonstrated that compound **85** selectively targeted cancer cells while exhibiting lower toxicity toward normal fibroblasts. The compound showed pronounced antiproliferative activity against MDA-MB-231 and MCF-7 breast cancer cell lines, surpassing that of DOX. Flow cytometric analysis revealed marked S-phase cell cycle arrest and substantial induction of early apoptosis. These findings were supported by a ~5.7-fold upregulation of Bax, a ~4.5-fold increase in caspase-3 levels, and an approximately 2-fold reduction in Bcl-2 expression, indicating activation of the mitochondrial apoptotic pathway.

Eissa, I.H. et al. (2025) [62] synthesized 2,3-dihydro-1,3,4-thiadiazole and sulfonamide derivatives. The thiadiazole scaffold was chosen due to its reported anticancer activity and ability to inhibit both kinases and CA isoforms such as hCA IX/XII. Its structural versatility also allows modifications that can enhance potency and target selectivity, making it suitable for dual-target inhibitor design. The cytotoxic effects of the synthesized thiadiazole-sulfonamide derivatives were investigated using the breast cancer cell lines MDA-MB-231 and MCF-7. Normal Vero cells were also included to evaluate the selectivity of their cytotoxic activity. Compound **86** (Figure 12) was the most active derivative, displaying IC_50_ values of 5.78 ± 0.3 µM against MDA-MB-231 cells and 8.05 ± 0.5 µM against MCF-7 cells, with greater potency than the reference drug sorafenib (IC_50_ = 7.64 ± 0.4 µM) (Table 16). Notably, compound **86** exhibited excellent selectivity toward cancer cells, with an IC_50_ value of 313.08 ± 2.96 µM in normal Vero cells, indicating low toxicity toward healthy cells. Compound **86** exhibited strong dual-targeting activity, achieving an IC_50_ value of 5.92 ± 0.69 nM against EGFR, which was lower than the reference drug, erlotinib (IC_50_ = 7.74 ± 0.59 nM). Compound **86** also exhibited potent inhibitory activity against CA IX, with an IC_50_ value of 63 ± 4 nM. This activity surpassed the reference inhibitor AAZ (IC_50_ = 86 ± 5 nM), indicating its strong ability to target this tumor-associated isoform. Compound **86** markedly reduced the viability of MDA-MB-231 breast cancer cells, decreasing viable cells to 5.92% compared with 95.48% in untreated controls. It strongly induced apoptosis, with early and late apoptosis reaching 38.73% and 54.60%, respectively, while necrosis remained minimal (0.73%). Overall, the results indicate that compound **86** exerts potent cytotoxic effects primarily through apoptosis rather than necrotic cell death. Apoptosis induction was further supported by a 13.97-fold elevation in the BAX/Bcl-2 ratio, along with increased caspase expression and cell cycle arrest at the G1 phase, confirming the activation of apoptotic pathways. These findings highlight the promise of thiadiazole-sulfonamide derivatives, especially compound **86**, as potential dual-targeted anticancer agents. Further studies should also explore its effectiveness in combination therapy approaches.

Eissa, I.H. et al. (2025) [63] also synthesized a series of novel sulfamoylphenyl-dihydro-thiadiazole derivatives. Compound **87** (Figure 12) was tested for the inhibitory activities (IC_50_ values) against EGFR, hCA IX, and hCA XII. Compound **87** showed excellent inhibitory effect against EGFR with IC_50_ value of 10.12 ± 0.29 nM. Erlotinib was the reference drug with IC_50_ = 7.74 ± 0. 59 nM. For hCA IX and hCA XII, compound **87** demonstrated promising inhibitory activity with IC_50_ values of 79 ± 0.3 nM and 58 ± 0.3 nM, respectively. AAZ was the reference compound with IC_50_ = 86 ± 0.5 nM against hCA IX and IC_50_ = 33 ± 0.2 nM against hCA XII. Cytotoxicity of compound **87** was tested against the MDA-231 and MCF-7 cancer cell lines. Compound **87** showed promising cytotoxic activity with IC_50_ values of 16.13 ± 1.2 µM against MDA-231 and 22.57 ± 1.5 µM against MCF-7, compared with AAZ (IC_50_ = 7.64 ± 0.4 against MDA-231 and 7.26 ± 0.3 µM against MCF-7). A significant advantage of compound **87** lies in its markedly lower cytotoxicity toward non-cancerous Vero cells, with an IC_50_ value of 148.32 ± 1.22 µM, compared with AAZ with IC_50_ of 77.57 ± 0.57 µM (Table 16). Compound **87** was evaluated for its effect on the expression of key apoptotic markers, including BAX, Bcl-2, the BAX/Bcl-2 ratio, caspase-8, and caspase-9, in MDA-MB-231 cells using real-time quantitative PCR. Results showed that compound **87** significantly upregulated the pro-apoptotic BAX gene (3.06 ± 0.18-fold) while simultaneously downregulating the anti-apoptotic Bcl-2 gene (0.55 ± 0.05-fold) compared to untreated control cells (set at 1.00-fold). This shift in the balance of BAX and Bcl-2 resulted in a marked increase in the BAX/Bcl-2 ratio (5.56 ± 0.69-fold), being a critical determinant of mitochondrial-mediated apoptosis. Also, compound **87** induced the activation of both Caspase-8 (2.37 ± 0.07-fold) and Caspase-9 (2.26 ± 0.15-fold). They tested compound’s effects on cell viability, apoptosis (early and late stages), and necrosis in MDA-MB-231 cells, compared to untreated control cells. In untreated MDA-MB-231 cells, the majority remained viable (95.48%), with minimal evidence of apoptosis (early: 3.84%, late: 0.32%) or necrosis (0.45%). Treatment with compound **87** markedly reduced cell viability to 19.20%, indicating strong cytotoxic activity. This effect was associated with a clear increase in both early (22.50%) and late apoptosis (58.27%), suggesting that cell death in MDA-MB-231 cells mainly occurs through an apoptotic mechanism. Necrosis was nearly absent (0.03%) following treatment, indicating that compound **87** promotes apoptotic pathways rather than uncontrolled cell death. The effect of compound **87** on the cell cycle distribution of MDA-MB-231 cells was evaluated. Results showed that compound **87** might exert its anti-proliferative effects by targeting proteins involved in G1/S transition (49.10% in treated cells vs. 44.98% in controls). In silico toxicological studies indicated a favorable safety profile, with low irritancy and acceptable rat oral LD_50_ (15.81 mg/kg) and carcinogenic potency (TD_50_ = 36.95). Molecular docking studies of compound **87** showed favorable binding within hCA IX and XII, mainly through coordination of the sulfonamide group with the Zn^2+^ ion, along with additional hydrogen bonding and hydrophobic interactions. Similarly, docking into EGFR revealed multiple hydrogen bonds and hydrophobic contacts, supporting strong binding within the active site. Compound **87** is a promising dual inhibitor targeting EGFR and hCA IX and hCA XII, demonstrating potent anticancer activity and a favorable safety profile. By structurally merging a zinc-binding sulfamoyl motif with a versatile dihydro-thiadiazole core, the compound achieves potent in vitro anti-cancer activity. Compound **87** significantly reduces cell viability and triggers apoptosis in MDA-MB-231 breast cancer cells, indicating its therapeutic efficacy in aggressive, hypoxic peripheral tumors where both EGFR and CA IX/XII are frequently overexpressed. SAR analysis indicated that incorporation of an ethoxy substituent into the sulfamoylphenyl–dihydrothiadiazole ketone scaffold resulted in a decrease in biological activity. The ethoxy-substituted derivative showed weaker inhibition of both EGFR and CA-IX, accompanied by reduced anticancer potency against MDA-MB-231 and MCF-7 cell lines, as reflected by higher IC_50_ values compared with compound **87**.

In their recent study, Elkaeed, E.B. et al. (2025) [64], synthesized a series of novel sulfamoylphenyl-dihydro-thiadiazole derivatives. Compound **88** (Figure 12) demonstrated potent dual inhibition of CA IX and EGFR, with IC_50_ values of 0.046 ± 0.007 µM and 0.059 ± 0.009 µM, respectively. Considering CA IX inhibition, the IC_50_ value closely matched AAZ (0.039 ± 0.005 µM) while its EGFR inhibition was slightly weaker than erlotinib (0.027 µM) (Table 16). Cytotoxicity assessment of compound **88** across a panel of human cancer cell lines HePG-2, MCF-7, HCT-116, PC-3, and MDA-MB-231 revealed a moderate to good anticancer profile, particularly in MDA-MB-231 (IC_50_ = 18.15 ± 1.4 µM) and MCF-7 (25.39 ± 1.7 µM) cells. Compound **88** showed a relatively high IC_50_ in WI-38 normal fibroblasts (72.46 ± 3.6 µM), suggesting a favorable therapeutic window and reduced off-target toxicity. Cell cycle analysis in MDA-MB-231 cells treated with compound **88** revealed a significant G0/G1 phase arrest, with a marked increase in the G0/G1 population from 37.45% (control) to 63.56%. Annexin V/PI staining of MDA-MB-231 cells treated with compound **88** revealed a substantial increase in apoptosis, with early apoptotic cells rising to 76.73%, compared to just 0.10% in untreated controls. The pro-apoptotic activity of compound 84 was further validated, with results revealing upregulation of Bax and caspase-3 (3.7-fold increase) and downregulation of Bcl-2 (1.97-fold decrease), consistent with activation of the intrinsic apoptotic pathway. Molecular dynamics simulations showed that compound **88** forms stable complexes with EGFR and CA IX.

### 3.2. Anilinoquinazoline-Sulfonamide Derivatives

**Figure 13:**
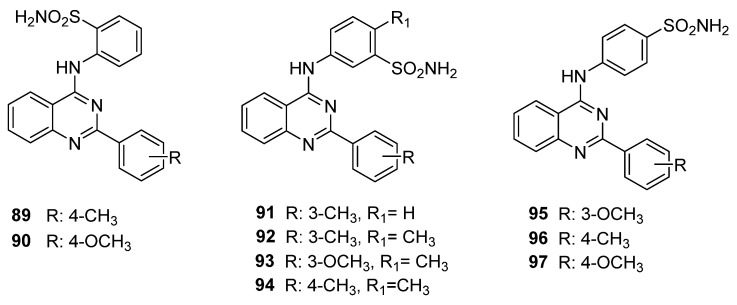
Chemical structures of anilinoquinazoline-sulfonamide derivatives (**89**–**97**) [65].

Eldehna, W.M. et al. (2024) [65] synthesized a new set of anilinoquinazoline-sulfonamide derivatives. 4-anilinoquinazoline scaffolds were chosen because they are well-known privileged structures with strong anticancer activity. They can effectively inhibit both CAs and EGFR. Their clinical success in drugs like erlotinib, gefitinib, and vandetanib further supports their ability to modulate cancer-related pathways. Overall, these scaffolds were selected as dual pharmacophores for targeting both CA enzymes and EGFR in cancer therapy. Compounds were examined for their inhibitory activity against the hCA I, II, IX and XII isoforms by the stopped-flow assay. AAZ was the reference drug (K_I_ = 250.0 nM against hCA I, 12.0 nM against hCA II, 25.0 nM against hCA IX and 5.7 nM against hCA XII). Among the tested isoforms, the hCA I isoform was the least affected, with K_I_ values of compounds **89**–**97** (Figure 13) ranging from 2.292 to 78.33 nM. Compound **96** was the best hCA I inhibitor with K_I_ values of 2.292 nM. Compounds **89**, **90** displayed the highest Ki values with 71.75 and 78.33 nM, respectively. The results indicate that compound **96**, bearing *para*-sulfonamide, demonstrated the best inhibition of hCA I. Compounds exhibited varying degrees of inhibition toward the hCA II isoform with KI values ranging from 4656 to 93.8 nM. Compounds **91**–**94** and **95**–**97**, bearing *meta* and *para* sulfonamide moiety effectively inhibited the hCA II isoform in the range: 224.1–912.2 nM and 93.8–396.8 nM, respectively, whereas *ortho* sulfonamide bearing compounds **89**, **90** displayed weak inhibitory activity (K_I_ = 4571–4656 nM). Compound **94**, with para sulfonamide and 3-methoxy substitution, was the most potent with K_I_ = 93.8 nM. Compounds **91**–**94** and **95**–**97**, demonstrated potent inhibition of the hCA IX isoform within K_I_ range from 44.0–404.2 nM and 38.4–183.4 nM, respectively. Compound **89**, **90**, containing ortho sulfonamide moiety, exhibited weak inhibitory activity, with K_I_ values of 4225 and 4256 nM, respectively. Compound **95** was the most potent inhibitor with K_I_ = 38.4 nM, with *para* sulfonamide and 3-methoxy moiety. Compounds were able to inhibit hCA XII with K_I_ values ranging from 9041 to 11.2 nM. Compounds **89**, **90** demonstrated the lowest inhibitory effect with K_I_ values of 9041 and 8308 nM, respectively. Compounds **91**–**97** showed potent results with K_I_ values ranging from 41.6 to 11.2 nM. Compound **97**, with *para* sulfonamide and 4-methoxy substitution, was the best one (K_I_ = 11.2 nM) (Table 17). SI was calculated for each compound and presented and exhibited an excellent selectivity profile toward hCA IX over hCA I, with compounds **94**, **95**, and **97** exhibiting the highest SI values of 159, 101.3, and 61, respectively. Compounds **89**–**97** were screened for anticancer activity at a dose of 10 μM using the SRB protocol against 59 cancer cell lines representing nine cancer types, including leukemia, non-small cell (NSC) lung, colon, CNS, melanoma, ovarian, renal, prostate and breast cancers. These compounds showed broad anticancer activity across multiple cell lines, as indicated by mean GI% values ranging from 23% to 72%, and they effectively inhibited the growth of most tested cell lines, with GI% values between 20% and 180%. Compound **92** was the most potent antiproliferative agent, with a mean GI% of 72%, and exhibited broad growth inhibition, suppressing 57 cancer cell lines across all subpanels. Compound **90**–**92** were selected for further examination at five doses level (0.01–100 μM) by NCI. Compound **90** showed remarkable anticancer activity against tested cancer cell lines, with GI_50_ values ranging from 1.81 to 9.42 μM across 44 tumor cell lines. Compounds **89**–**97** were evaluated for their inhibitory effect against EGFR, with erlotinib being the reference drug (IC_50_ = 80 ± 2.0 nM). Compounds showed good to excellent IC_50_ values ranging from 51.2 ± 0.97 to 211.9 ± 7.3 nM. Compound **95**, 2-(3-methoxyphenyl) quinazoline bearing *para* sulfanilamide, exhibited the most potent results (IC_50_ = 51.2 ± 0.97 nM). Compounds **90** and **93** possessed remarkably activities with IC_50_ values of 82.7 ± 1.7 and 84.4 ± 1.25 nM, respectively. Methoxy-substituted derivatives were generally more potent than methyl analogues, with the *meta*-methoxy position being the most favorable. *Para*-sulfonamide derivatives tended to show higher activity than *meta* and *ortho* analogues, although some exceptions were observed. Compound **95** showed strong binding within the hCA IX active site through sulfonamide coordination with Zn^2+^, supported by hydrogen bonding and hydrophobic interactions with key residues. Docking studies showed that compound **94** fits well within the EGFR active site, forming multiple hydrogen bonds and hydrophobic interactions, with the methoxyphenyl group contributing to its enhanced binding affinity. Compound **95** emerged as the most potent dual inhibitor, with IC_50_ values comparable to the reference drugs. The results indicate that para-positioned sulfonamide is more favorable for inhibition compared to ortho and meta substitutions, likely due to its optimal orientation for Zn^2+^ coordination within the CA active site. In addition, the presence of a methoxy group proved more beneficial than a methyl substituent, which may be attributed to its ability to enhance electronic effects and participate in additional polar interactions, thereby improving binding affinity and overall dual inhibitory activity. Kinase selectivity profiling against 40 kinases representing the diverse human protein kinase families proved that compound 90 is a selective EFGR inhibitor.

### 3.3. 3-Cyanopyridin-2-One Sulfonamide Derivatives

**Figure 14:**
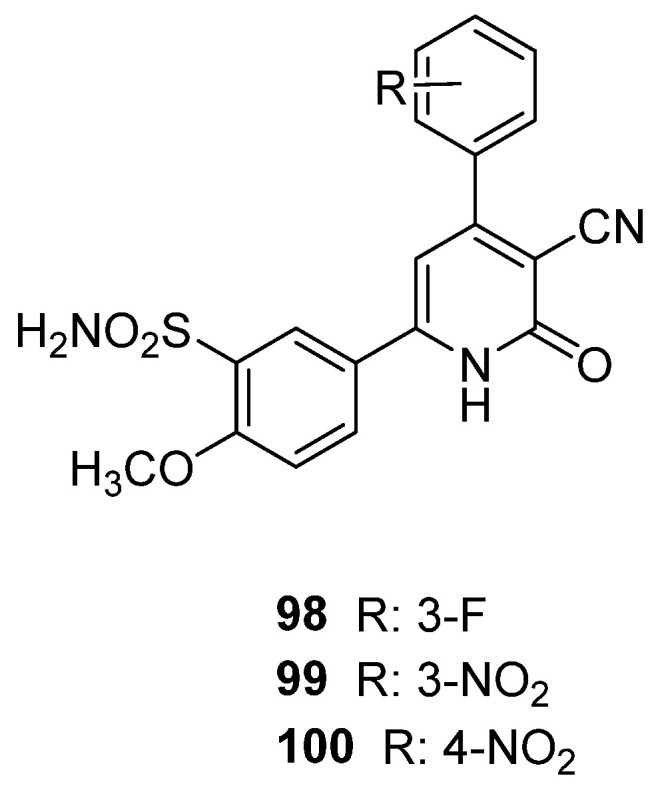
Chemical structures of 3-cyanopyridin-2-one sulfonamide derivatives (**98**–**100**) [66].

Shaldam, M.A. et al. (2023) [66] constructed a novel class of 3-cyanopyridin-2-one sulfonamide derivatives. Cyanopyridine derivatives are known to inhibit key targets such as EGFR and CAs, making them suitable for dual-target drug design. In addition, the presence of the cyano group and pyridone ring provides structural versatility for further functionalization, particularly with sulfonamide moieties, enabling strong Zn^2+^ binding in CA active sites. Compounds **98**–**100** (Figure 14) were tested for their inhibitory effects against hCA I, II and IX. Unfortunately, none of the tested compounds (K_I_ > 100 µM) did not inhibit the hCA isoforms I, II, and IX. Considering EGRF inhibition, compounds showed good to excellent results with IC_50_ values of 95.1 ± 2.8 to 113.0 ± 7.5 µM. Compound **97**, featuring 3-F substitution, was the most potent (IC_50_ = 95.1 ± 2.8 nM), compared with erlotinib (IC_50_ = 78.6 ± 2.4 nM). It was observed that positioning the NO_2_ group at the *para* position (compound **100**), compared to the *meta* position (compound **99**), slightly enhanced the inhibitory effect, with IC_50_ values of 104.2 ± 4.1 and 113.0 ± 7.5 nM, respectively. Antiproliferative activity was evaluated against HeLa and MDA-MB-231 cells, using the MTT assay. Compounds **98**–**100** showed excellent activities against MDA-MB-231 with IC_50_ values of 0.19 ± 0.01, 0.22 ± 0.01 and 0.13 ± 0.01µM, respectively compared with staurosporine (IC_50_ = 0.21 ± 0.01 µM) and 5-FU (IC_50_ = 0.49 ± 0.02 µM). Compound **100** was the most potent, bearing 4-NO_2_ group. Considering HeLa cells, compounds **98**–**100** demonstrated excellent inhibitory effect with IC_50_ values of 0.14 ± 0.01, 0.16 ± 0.01 and 0.12 ± 0.01 µM, respectively compared with staurosporine (IC_50_ = 0.39 ± 0.02 µM) and 5-FU (IC_50_ = 0.52 ± 0.03 µM) (Table 18). Once again, compound **100** had the best inhibitory activity. Compounds **98** and **100** were chosen to better understand their cellular mechanisms of action. The DNA concentration was measured using flow cytometry after HeLa cells were treated with DMSO (control). Both possessed comparable cell-cycle arrest patterns. Compared with 1.82% in untreated HeLa cells, cells treated with compound **98** and compound **100** showed an increase in the proportion of cells in the pre-G1 phase (31.94% and 38.17%, respectively). Treatment with compounds **98** and **100** markedly increased apoptosis in HeLa cells, raising both early and late apoptotic populations compared to untreated cells. Overall, total apoptosis was enhanced by about 41- and 35-fold for compound **98** and **100**, respectively, indicating strong pro-apoptotic activity. Molecular docking studies of compound **98** within the EGFR active site yielded a docking score of −9.7 kcal/mol. Compounds **98**–**100** demonstrated a complete loss of hCA inhibitory activity while completely retaining its potency against EGFR. This shift in selectivity highlights a critical SAR driven by substitution topology and steric hindrance. The *meta* relationship between the zinc-binding sulfonamide group and the bulky dihydropyridine core, compounded by the presence of an *ortho*-methoxy group, breaks the linear geometry required for a tail-approach molecule to penetrate the narrow, conical catalytic cavity of hCAs. Consequently, this derivative transitions from a dual inhibitor to a highly selective EGFR agent, providing a clear structural blueprint for decoupling kinase inhibition from metalloenzyme cross-reactivity.

### 3.4. Quinazoline Derivatives Bearing a Benzene-Sulfonamide Moiety

**Figure 15:**
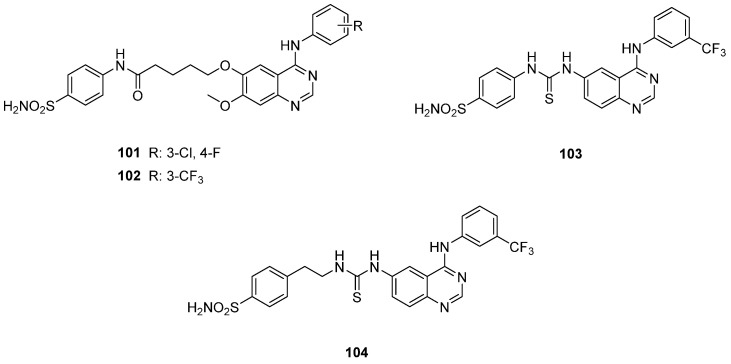
Chemical structures of quinazoline derivatives bearing a benzene-sulfonamide moiety (**101**–**104**) [67].

Zhang, B. et al. (2021) [67] developed and analyzed a new set of quinazoline derivatives bearing a benzene-sulfonamide moiety. Quinazoline was chosen as a well-known EGFR-inhibiting anticancer scaffold, while sulfonamide was included as a privileged group with established CA inhibitory activity. Their combination was designed to achieve dual anticancer action through EGFR and CA inhibition. Compounds **101**–**104** (Figure 15) were tested for their inhibitory effect against hCA II and IX isoforms. AAZ was used as a reference drug with IC_50_ values of 45.1 ± 7.4 nM against hCA II 87.2 ± 9.6 nM against hCA IX. Compounds **101**–**104** showed moderate to low inhibitory activities against hCA II with IC_50_ values ranging between 241.5 ± 43.7 to 526.2 ± 88.3 nM Compound **104** was the most potent (IC_50_ = 241.5 ± 43.7 nM. Considering hCA IX inhibition compounds showed moderate results with compound **102** being the most potent with IC_50_ value of 115.0 ± 16.8 nM and showed the highest SI (2.4) as well. Compound **104** showed stronger CA (CAII and CAIX) inhibitory activity than **103**, which may be attributed to a longer carbon chain that enhanced the binding affinity of compound **104** to CA. The inhibitory activity of compounds **101**–**104** against EGFR_WT_ and EGFR_T790M_ were evaluated using a well-established HTRF KinEASE-TK assay. Gefitinib (IC_50_ = 17.1 ± 4.2 nM against EGFR_WT_ and 378.4 ± 56.8 nM against EGFR_T790M_) and osimertinib (IC_50_ = 58.2 ± 12.6 nM against EGFR_WT_ and 8.1 ± 2.2 nM against EGFR_T790M_) were used as reference drugs. Compounds exhibited moderate inhibitory activities against EGFR_WT_ (with IC_50_ values ranging from 13.7 ± 4.0 to 51.2 ± 10.4 nM), in which compound **101** (IC_50_ = 13.7 ± 4.0 nM) suppressed EGFR_WT_ more potently than gefitinib. Compound **98** exhibited excellent inhibitory effect on EGFR_T790M_, with an IC_50_ value of 9.2 nM, being 41 times as effective as gefitinib. Compounds **101**–**104** were evaluated for their antiproliferation activity against human epidermoid carcinoma cells (A431) and non-small cell lung cancer cells (A549 and H1975), using MTT assay. Gefitinib, osimertinib and erlotinib were used as standards. All of the tested compounds showed excellent inhibitory effect against A549 cancer cells with IC_50_ values ranging between 9.05 ± 0.31 to 10.14 ± 0.24 μM, compared with gefitinib (IC_50_ = 15.59 ± 1.03 μM) and erlotinib (IC_50_ = 16.43 ± 0.96 μM). Compound **104** was the most potent, bearing -CF_3_ substitution, with IC_50_ value of 9.05 ± 0.31 μM. Considering A431 cancer cells, compounds exhibited good inhibitory effects with IC_50_ values ranging from 3.91 ± 0.23 to 21.68 ± 0.16 μM, compared with gefitinib (IC_50_ = 8.37 ± 0.46 μM), erlotinib (IC_50_ = 11.85 ± 0.69 μM) and osimertinib (IC_50_ = 5.32 ± 0.43 μM). Compound **101**, with 3-CF_3_ and 4-Cl substitutions, was the most promising one (IC_50_ = 3.91 ± 0.23 μM). Compounds also demonstrated excellent inhibitory activities against H1975 cancer cells with IC_50_ values ranging between 1.94 ± 0.14 to 7.59 ± 0.21 μM, compared with gefitinib (IC_50_ = 10.78 ± 0.45 μM), erlotinib (IC_50_ = 13.12 ± 0.97 μM) and osimertinib (IC_50_ = 0.98 ± 0.01 μM) (Table 19). Compound **102** showed the best inhibitory activity (IC_50_ = 1.94 ± 0.14 μM), with 3-CF_3_ substitution. The cytotoxicity of compound **102** was also evaluated in normal human liver LO2 cells to assess the safety profile of the synthesized anticancer agent. Gefitinib and osimertinib were also used as reference compounds. Results showed that gefitinib exhibited moderate cytotoxicity (IC_50_ = 33.17 μM), whereas both compound **102** and osimertinib showed minimal toxicity (IC_50_ > 40 μM), suggesting a favorable safety profile toward normal liver cells. Apoptotic analysis of compound **102** against H1975 cells was carried out by an Annexin VFITC/PI assay and gefitinib was the standard. Overall, the results verified that compound **102** successfully induced apoptosis in H1975 cells. Also, the effect of compound **102** on cell cycle arrest of H1975 cells was tested and results showed that compound **98** arrested the G2/M phase of the cell cycle in a dose dependent manner. Moreover, they tested inhibition of migration by wound healing assays in H1975 cell line. The migration rate decreased significantly with the increase of the concentration of compound **102**. After treatment with 1.0 μM of compound **102**, the migration rate decreased from 51.2% to 17.9%. The ability of compound **102** to selectively inhibit EGFR phosphorylation and its downstream signaling pathways (AKT and ERK) was evaluated using a Western blot assay. Results showed that after **102** treatment, p-EGFR, p-AKT and p-ERK expression levels were decreased obviously in a dose dependent manner. To evaluate the antiproliferative activity of compound **102** under hypoxic conditions, A549 and H1975 cells were exposed to varying concentrations of **102** for 72 h. Hypoxia was established by incubating the cells in an atmosphere containing 1% O_2_, 5% CO_2_, and 94% N_2_. Compound **102** demonstrated enhanced anticancer activity under hypoxic conditions, with roughly a threefold increase in A549 cells and a twofold improvement in H1975 cells compared to normoxia. Molecular docking studies indicated that compound **102** effectively binds to EGFR_WT_, EGFR_T790M_, and CAIX, with enhanced interactions (particularly additional hydrogen bonding) contributing to stronger binding affinity, especially toward CAIX and the mutant EGFR form. Compound **102** showed the best dual inhibitor profile driven from two critical structural features: linker length/flexibility and the presence of the methoxy group on the quinazoline core. Structure of compound **102** satisfies the strict geometric constraints of the hCA IX catalytic cavity while simultaneously enhancing hydrophobic fit and reducing structural strain within the EGFR active site.

## 4. Conclusions

**Figure 16:**
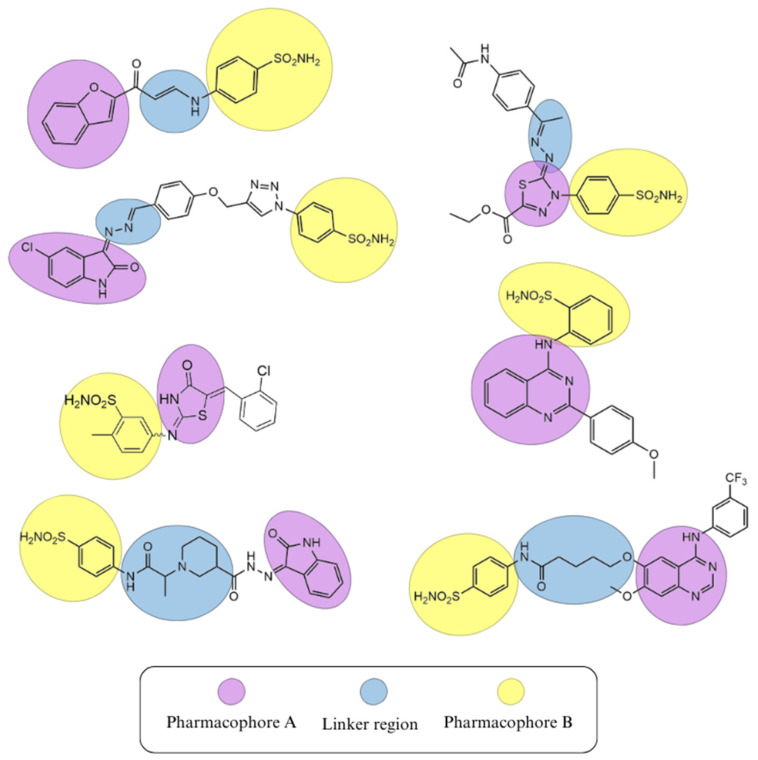
SAR-driven structural requirements for dual target CA IX and VEGFR-2 or EGFR inhibition.

Dual targeting of CA IX and oncogenic signaling pathways like VEGFR-2 and EGFR, represents a promising approach in anticancer therapy. CA IX plays a crucial role in regulating pH and facilitating the adaptation of cancer cells to hypoxic conditions, thereby enhancing their survival and aggressiveness. Moreover, VEGFR-2 and EGFR are critically involved in angiogenesis, cell proliferation, and the transmission of survival signals. The simultaneous targeting of these mechanisms allows modulation at multiple levels of cancer progression, enhancing therapeutic efficacy and limiting the development of resistance. It was found that several potent inhibitors exhibited good to excellent dual activity against VEGFR-2/EGFR and CA IX (Table 20). Compounds **2**, **3**, **9**, **14**, **23**, **28**, **47** and **65** exhibited excellent dual inhibitory effect against CA IX and VEGFR-2, with a couple of them lower than those of the reference compounds. All of these compounds feature a sulfonamide functional group, apart from compound **23**, containing coumarin and thiazole moieties. It was also observed that most of them feature the sulfonamide moiety linked to a benzene ring at the *para* position. Compound **28** was the only *N*-substituted sulfonamide, linked to cyclohexane. Also, four of the seven best compounds contained a halogen atom, with Cl and F being the most prevalent (compounds **9**, **14**, **23** and **47**). Most compounds contained either hydrazine or acetamide moieties as linkers within their structures. Finally, compounds feature at least one heterocycle ring, with triazole ring being the most favorable (compounds **9**, **28** and **47**). Considering dual CA IX and EGFR inhibition, compounds **85**, **90**, **93**, **95** and **102** showed excellent results. The compounds also featured a sulfonamide moiety linked to a benzene ring and contained a heterocyclic ring within their structure, with quinazoline being the most favorable (compounds **90**, **93**, **95** and **102**). Hydrazine and acetamide linkers were also present in several of these structures. Among these compounds, only compound **102** possessed an extended aliphatic chain as a linker. The SAR data of the most effective dual CA IX-VEGFR-2 and CA IX-EGFR, highlight a few essential design principles. To maintain strong potency against both targets, the molecules require an unhindered zinc-binding group, like a primary sulfonamide or a coumarin ring for CA IX affinity, combined with a privileged kinase-binding framework such as quinazoline or indole core. Most importantly, the best-performing compounds avoid directly fusing these pieces together. Instead, they utilize a flexible linker to separate the two domains, which prevents the bulky kinase inhibitor portion from physically blocking access to the narrow active site of CA IX (Figure 16). The compounds were evaluated for their antiproliferative activity against various cancer cell lines and demonstrated excellent results. The studied compounds were also evaluated for their effects on several apoptotic markers and demonstrated promising results. Also, molecular docking studies were conducted on the most promising compounds and revealed favorable binding interactions within the active sites of CA IX, VEGFR-2, and EGFR. Finally, their selectivity was assessed against other CA isoforms, and most compounds showed excellent selectivity for CA IX over the other isoforms, potentially reducing side effects. However, these studies did not evaluate the compounds in comparison with a combination of selective CA IX and EGFR inhibitors in cellular assays. Therefore, although the enzymatic profile appears promising, it remains unclear whether this single chemical entity provides a synergistic therapeutic advantage over the coadministration of two separate selective agents. However, it appears that compounds inhibiting both CA IX and VEGFR-2 share similar SAR characteristics with CA IX/EGFR inhibitors. This observation could facilitate the discovery and synthesis of molecules capable of acting as triple-target inhibitors. The ATP-binding pockets of VEGFR-2 and EGFR share several overlapping structural features, which may facilitate the development of triple-action inhibitors. Hybridization of a dual-acting kinase targeting scaffold, such as quinazoline and indole-based motifs, with a primary benzenesulfonamide moiety through an optimized, flexible linker could lead to a triple-targeted molecule. This strategy may effectively suppress the compensatory signaling pathways that tumors frequently activate in response to inhibition of a single kinase pathway.

## Figures and Tables

**Table 1:** CA inhibitors and general information on their current status.

Drug	Structure	Current Status	References
Acetazolamide	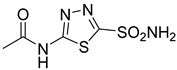	systemic CAI clinically used to treat glaucoma, edema and certain types of epilepsy	[26,27]
Methazolamide	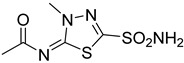	systemic CAI clinically used to treat glaucoma	[26,27]
SLC-0111	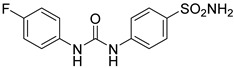	clinical trials as adjuvant agent in advanced solid tumors Phase Ib/II	[28]

**Table 2:** USFDA approved drugs targeting VEGFRs.

Drug	Structure	Year of Approval	References
Sorafenib	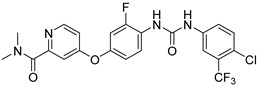	2005	Iyer, R. et al. [47]
Sunitinib	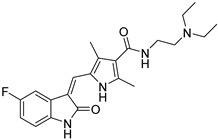	2006	Blumenthal, G.M. et al. [48]
Regorafenib	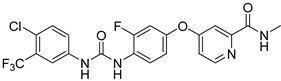	2012	Ettrich, T.J. et al. [49]
Lenvatinib	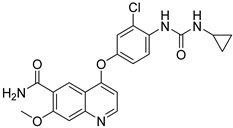	2018	Nair, A. et al. [50]
Fruquintinib	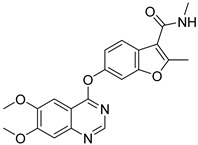	2023	Fusco, M.J. et al. [51]

**Table 3:** Bioassay results for compounds **1**, **2** and reference compounds [52].

Compound	hCA IX Inhibition IC_50_ (μM)	hCA I Inhibition IC_50_ (μM)	hCA II Inhibition IC_50_ (μM)	hCA XII Inhibition IC_50_ (μM)	VEGFR-2 IC_50_ (μM)
**1**	0.168 ± 0.05	0.290 ± 0.03	0.624 ± 0.07	0.154 ± 0.06	0.145 ± 0.02
**2**	0.077 ± 0.01	0.448 ± 0.02	0.340 ± 0.004	0.205 ± 0.06	0.069 ± 0.01
Reference: AAZ	0.056 ± 0.01	0.228 ± 0.03	0.134 ± 0.01	0.109 ± 0.06	-
Reference: Sorafenib	-	-	-	-	0.057 ± 0.01

**Table 4:** Bioassay results for compounds **1**, **2** and reference compounds [52].

Compound	Hela IC_50_ (µM)	HepG-2 IC_50_ (μM)	HCT-116 IC_50_ (μM)	MCF-7 IC_50_ (μM)	WI-38 IC_50_ (μM)
**1**	11.57 ± 0.9	10.90 ± 0.8	21.43 ± 1.5	13.40 ± 1.1	39.89 ± 2.4
**2**	14.11 ± 1.2	7.81 ± 0.4	19.50 ± 1.3	9.61 ± 0.7	54.18 ± 3.2
Reference: Doxorubicin (DOX)	5.57 ± 0.4	4.50 ± 0.2	5.23 ± 0.3	4.17 ± 0.2	6.72 ± 0.5

**Table 5:** Bioassay results for compounds **3**–**8** and reference compounds [53].

Compound	hCA IX Inhibition (K_I_, nM)	hCA I Inhibition (K_I_, nM)	hCA II Inhibition (K_I_, nM)	hCA XII Inhibition (K_I_, nM)	SR_I/IX_	SR_I/XII_	SR_II/IX_	SR_II/XII_	VEGFR-2 Inhibition (IC_50_, μM)
**3**	35	6712	7.5	1.3	191.8	5163	0.21	5.7	0.058 ± 0.001
**4**	137.0	52,470	980.8	221.1	382.9	237.3	7.15	4.43	1.024 ± 0.220
**5**	171.0	13,620	81.8	104.8	79.6	129.9	0.47	0.78	0.155 ± 0.010
**6**	881.2	>100	-	533.1	113.4	187.5	-	-	
**7**	882.2	81,020	-	514.3	91.8	157.5	-	-	
**8**	754.2	97,340	-	912.7	129.0	106.6	-	-	
Reference: Acetazolamide	25	250.0	12.0	5.7	10.0	43.8	0.48	2.1	
Reference: Pazopanib	-	-	-	-	-				0.059

**Table 6:** Bioassay results for compounds **3**–**5** and reference compounds [53].

Compound	MCF-7 (IC_50_, μΜ)		PC-3 (IC_50_, μΜ)	
	(Breast Cancer)		(Prostate Cancer)	
	Under Normoxia	Under Hypoxia	Under Normoxia	Under Hypoxia
**3**	4.87 ± 0.57	3.65 ± 0.40	7.58 ± 0.45	5.62 ± 0.92
**4**	73.55 ± 4.50	17.56 ± 0.78	53.44 ± 1.15	34.48 ± 2.23
**5**	10.19 ± 0.76	9.03 ± 0.91	21.76 ± 2.29	14.09 ± 0.71
Reference: DOX	1.47 ± 0.14	9.42 ± 0.65	2.60 ± 0.12	6.06 ± 0.74

**Table 7:** Bioassay results for compounds **9**–**12** and reference compounds [54].

Compound	hCA I Inhibition (K_I_, nM)	hCA II Inhibition (K_I_, nM)	hCA IX Inhibition (K_I_, nM)	hCA XII Inhibition (K_I_, nM)	SI (hCA II/hCA IX)	SI (hCA II/hCA XII)	VEGFR-2 Inhibition (IC_50_, μM)
**9**	5573 ± 222	182.60 ± 7.30	27.30 ± 1.10	221 ± 8.40	6.69	0.83	0.06 ± 0.004
**10**	>10,000	709.40 ± 17.30	180.20 ± 7.20	72.90 ± 2.80	3.94	9.73	0.17 ± 0.011
**11**	>10,000	292.80 ± 10.50	31.50 ± 1.10	386.90 ± 12.10	9.30	0.76	0.12 ± 0.007
**12**	>10,000	854.80 ± 27.20	37.20 ± 1.50	726.90 ± 14.10	22.98	1.18	0.24 ± 0.015
Reference: AAZ	250.0 ± 10	12.10 ± 0.48	25.80 ± 1.02	5.70 ± 0.22	0.47	2.12	-
Reference: Sunitinib	-	-	-	-	-	-	0.09 ± 0.002

**Table 8:** Bioassay results for compounds **7**–**10** and reference compounds [54].

Compound	MDA-MB 231 (IC_50_, μM) and SI Values	MCF-7 (IC_50_, μM) and SI Values	A549 (IC_50_, μM) and SI Values	Caco-2 (IC_50_, μM) and SI Values	WI-38 (IC_50_, μM)
**9**	5.83 ± 0.25, 13.77 respectively	22.81 ± 0.71, 3.52 respectively	39.47 ± 1.14, 2.03 respectively	18.74 ± 0.52, 4.28 respectively	80.30 ± 2.59
**10**	11.38 ± 0.49, 3.01 respectively	7.45 ± 0.23, 4.60 respectively	12.49 ± 0.36, 2.74 respectively	4.56 ± 0.13, 7.51 respectively	34.28 ± 1.11
**11**	1.30 ± 0.06, 24.20 respectively	2.28 ± 0.07, 13.79 respectively	9.53 ± 0.28, 3.30 respectively	2.07 ± 0.06, 15.19 respectively	31.46 ± 1.01
**12**	19.38 ± 0.83, 2.15 respectively	20.69 ± 0.64, 2.01 respectively	36.96 ± 1.07, 1.12 respectively	14.40 ± 0.40, 2.89 respectively	41.70 ± 1.34
Reference: 5-FU	18.93 ± 0.86, 3.01 respectively	18.09 ± 0.56, 3.15 respectively	47.89 ± 1.38, 1.19 respectively	3.19 ± 0.09, 17.89 respectively	57.09 ± 1.84

**Table 9:** Bioassay results for compounds **13**–**18** and reference compounds [55].

Compound	CAIX IC_50_ (µM)	VEGFR-2 IC_50_ (µM)	MCF-7 IC_50_ (µM)
**13**	0.056 ± 0.003	0.095 ± 0.005	46.38 ± 3.43
**14**	0.035 ± 0.001	0.093 ± 0.005	21.32 ± 2.77
**15**	0.073 ± 0.004	0.116 ± 0.006	46.23 ± 2.93
**16**	0.059 ± 0.002	0.161 ± 0.008	30.86 ± 3.14
**17**	0.069 ± 0.003	0.254 ± 0.012	30.51 ± 2.90
**18**	0.041 ± 0.002	0.048 ± 0.002	22.33 ± 2.09
Reference: Dorzolamide	0.025 ± 0.001	-	-
Reference: AAZ	0.042 ± 0.002	-	-
Reference: Sorafenib	-	0.065 ± 0.003	8.39 ± 0.44
Reference: Staurosporine	-	-	11.2 ± 0.51

**Table 10:** Bioassay results for compounds **19**–**26** and reference compounds [56].

Compound	hCA I K_I_ (μM)	hCA II K_I_ (μM)	hCA IX K_I_ (μM)	hCA XII K_I_ (μM)	VEGFR-2 IC_50_ (µM)	PANC1	MCF7	PC3
**19**	>100	81.1	3.5	2.8	68.6 ± 1.80	1.07 ± 0.02	1.66 ± 0.01	6.10 ± 0.02
**20**	>100	63.4	4.5	3.7	-	-	-	-
**21**	>100	>100	6.7	6.5	-	-	-	-
**22**	>100	>100	3.7	5.2	34.8 ± 1.04	1.82 ± 0.00	3.01 ± 0.02	5.36 ± 0.03
**23**	>100	74.3	3.1	1.5	32.1 ± 1.10	1.04 ± 0.01	1.40 ± 0.00	5.03 ± 0.01
**24**	>100	97.7	6.1	3.8	-	-	-	-
**25**	>100	78.8	8.7	4.0	-	-	-	-
**26**	78.8	>100	6.9	9.1	-	-	-	-
Reference: AAZ	0.25	0.012	0.025	0.005	-	-	-	-
Reference: Sorafenib	>10	>10	8.1	>10	30.0 ± 0.09	-	-	-
Reference: Sunitinib	-	-	-	-	139	-	-	-
Reference: DOX	-	-	-	-	-	6.90 ± 0.09	4.17 ± 0.03	8.87 ± 0.02

**Table 11:** Bioassay results for compounds **19**–**26** and reference compounds [56].

Compound	SR_I/IX_	SR_II/IX_	SR_I/XII_	SR_II/XII_
**19**	28.5	23.1	35.7	28.9
**20**	22.2	14.0	27.0	17.1
**21**	14.9	14.9	15.3	15.3
**22**	27.0	27.0	19.2	19.2
**23**	33.3	24.7	66.6	49.5
**24**	16.3	16.0	26.3	25.7
**25**	11.4	9.0	25.0	19.7
**26**	11.4	14.4	8.6	10.9
Reference: AAZ	10.0	0.5	43.8	2.1

**Table 12:** Bioassay results for compounds **27**, **28** and reference compounds [57].

Compound	hCA IX IC_50_ (nM)	hCA XII IC_50_ (nM)	VEGFR-2 IC_50_ (µM)	A-549 IC_50_ (μM)	HepG-2 IC_50_ (μM)	MCF-7 IC_50_ (μM)	HCT-116 IC_50_ (μM)	RPE-1 IC_50_ (μM)
**27**	66	7.6	1.33 ± 0.10	20.45 ± 0.28	10.45 ± 0.13	20.31 ± 0.66	32.05 ± 0.42	82.58 ± 0.52
**28**	40	3.2	0.38 ± 0.14	19.81 ± 0.65	8.39 ± 0.20	21.15 ± 2.45	23.60 ± 0.22	87.22 ± 0.73
Reference: Sorafenib	-	-	0.43 ± 0.10	-	-	-	-	-
Reference: SLC-0111	53	4.8	-	-	-	-	-	-
Reference: DOX	-	-	-	-	13.76 ± 0.45	17.44 ± 0.46	-	-
Reference: Sunitinib	-	-	-	10.14 ± 0.50	-	-	9.67 ± 0.22	-

**Table 13:** Bioassay results for compounds **29**–**58** and reference compounds [58].

Compound	hCA I K_I_ (nM)	hCA II K_I_ (nM)	hCA IX K_I_ (nM)	hCA XII K_I_ (nM)	VEGFR-2 IC_50_ (nM)	MCF-7 IC_50_ (μM)	T47D IC_50_ (μM)	MCF-10A IC_50_ (μM)	SI
**29**	>10,000	384.1	75.9	47.5	-	-	-	-	-
**30**	>10,000	327.6	119.8	42.5	-	-	-	-	-
**31**	>10,000	241.1	100.2	68.7	271 ± 14.0	2.19 ± 0.09	2.97 ± 0.12	31.70 ± 1.2	14.47
**32**	>10,000	158.2	54.0	59.7	-	-	-	-	-
**33**	>10,000	158.1	92.0	52.9	183 ± 8.1	1.06 ± 0.04	2.34 ± 0.09	47.50 ± 1.79	44.81
**34**	>10,000	112.1	179.7	64.8	-	-	-	-	-
**35**	4899	99.0	32.1	36.9	-	-	-	-	-
**36**	5820	100.8	52.5	58.9	-	-	-	-	-
**37**	8261	152.4	69.7	48.5	-	-	-	-	-
**38**	9428	91.5	38.4	45.1	-	-	-	-	-
**39**	4189	56.0	48.6	15.8	-	-	-	-	-
**40**	>10,000	284.5	59.2	46.3	-	-	-	-	-
**41**	>10,000	169.7	28.0	42.0	-	-	-	-	-
**42**	>10,000	91.4	67.2	61.0	96.2 ± 2	10.9 ± 0.62	17.9 ± 0.78	77.21 ± 2.91	7.08
**43**	>10,000	126.9	84.2	95.4	541 ± 23.0	7.72 ± 0.32	11.6 ± 0.47	39.20 ± 1.1	5.08
**44**	>10,000	230.2	63.7	36.4	-	-	-	-	-
**45**	>10,000	72.4	81.7	41.1	717 ± 30.0	6.86 ± 0.28	18.7 ± 0.75	62.02 ± 2.34	9.04
**46**	>10,000	547.2	125.7	81.2	-	-	-	-	-
**47**	2251	25.2	8.3	4.7	26.3 ± 0.4	0.66 ± 0.04	4.51 ± 0.2	25.58 ± 0.97	38.76
**48**	2694	64.1	30.4	8.2	>1000	19.0 ± 1.08	23.9 ± 1.04	83.06 ± 3.14	4.37
**49**	1943	84.6	38.1	12.9	318 ± 13	2.17 ± 0.09	2.84 ± 0.11	64.94 ± 2.45	29.93
**50**	5108	13.6	5.2	6.9	-	-	-	-	-
**51**	3872	29.4	24.9	5.6	-	-	-	-	-
**52**	5419	16.9	19.3	9.7	-	-	-	-	-
**53**	39.8	3.4	2.2	10.4	-	-	-	-	-
**54**	61.8	20.1	5.4	12.7	-	-	-	-	-
**55**	81.4	41.7	6.2	25.8	-	-	-	-	-
**56**	100.7	35.8	9.5	4.9	-	-	-	-	-
**57**	82.7	0.9	17.1	3.2	-	-	-	-	-
**58**	152.7	16.4	23.3	12.9	-	-	-	-	-
Reference: AAZ	250.0	12.0	25.0	5.7	-	-	-	-	-
Reference: Sunitinib	-	-	-	-	39.7 ± 2	-	-	-	-
Reference: Staurosporine	-	-	-	-	-	3.18 ± 0.18	7.12 ± 0.31	-	-

**Table 14:** Bioassay results for compounds **59**–**77** and reference compounds [59].

Compound	hCA I K_I_ (nM)	hCA II K_I_ (nM)	hCA IX K_I_ (nM)	hCA XII K_I_ (nM)	VEGFR-2 IC_50_ (nM)	MDA-MB-231 IC_50_ (μM)	MCF-7 IC_50_ (μM)
**59**	82.1	32.1	52.8	13.5	-	-	-
**60**	62.3	20.3	35.4	0.93	-	-	-
**61**	115.6	0.75	22.1	19.7	-	-	-
**62**	136.9	58.2	65.9	29.8	-	-	-
**63**	99.0	18.4	15.8	20.1	-	-	-
**64**	64.2	2.3	19.0	9.7	-	-	-
**65**	75.3	16.1	3.6	16.7	204 ± 9	4.083 ± 0.175	9.997 ± 0.364
**66**	42.1	5.8	5.1	4.8	-	-	-
**67**	102.8	43.7	9.8	39.2	-	-	-
**68**	25.1	22.9	4.2	28.0	358 ± 16	7.204 ± 0.308	11.345 ± 0.413
**69**	97.6	30.8	29.4	58.4	-	-	-
**70**	71.2	25.1	33.5	21.3	-	-	-
**71**	253.1	39.5	21.4	66.9	-	-	-
**72**	205.3	42.0	9.8	61.2	-	-	-
**73**	369.8	62.8	19.2	51.7	-	-	-
**74**	62.4	15.0	24.8	20.1	-	-	-
**75**	35.5	19.8	6.2	3.8	811 ± 36	18.853 ± 0.807	29.292 ± 1.067
**76**	91.4	0.8	14.3	9.9	-	-	-
**77**	132.8	25.9	27.9	43.1	-	-	-
Reference: AAZ	250	12	25	5.7	-	-	-
Reference: Sorafenib	-	-	-	-	41 ± 2	8.704 ± 0.372	5.167 ± 0.188
Reference: 5-FU	-	-	-	-	-	-	-

**Table 15:** Bioassay results for compounds **78**–**84** and reference compounds [60].

Compound	hCA I K_I_ (μM)	hCA II K_I_ (μM)	hCA IX K_I_ (μM)	VEGFR-2 IC_50_ (nM)	T47D IC_50_ (μM)
**78**	>100	>100	>100	56.70 ± 0.72	5.45 ± 0.24
**79**	-	-	-	-	24.13 ± 1.08
**80**	>100	>100	>100	63.40 ± 0.72	1.83 ± 0.08
**81**	>100	>100	>100	30.10 ± 0.31	10.40 ± 0.47
**82**	-	-	-	-	11.58 ± 0.52
**83**	>100	>100	>100	23.10 ± 0.41	3.59 ± 0.16
**84**	-	-	-	-	16.52 ± 0.74
Reference: AAZ	0.25	0.012	0.026	-	-
Reference: DOX	-	-		-	2.26 ± 0.10
Reference: Sorafenib	-	-	-	29.70 ± 0.17	-

**Table 16:** Bioassay results for compounds **85**–**88** and reference compounds [61,62,63,64].

Compound	hCA IX IC_50_	hCA XII IC_50_ (nM)	EGFR IC_50_	MDA-231 IC_50_ (µM)	MCF-7 IC_50_ (µM)	WI-38 (µM)	Vero
**85**	0.039 μM	0.04 μM	0.05 μM	11.22 ± 1.0	22.04 ± 1.3	-	-
**86**	63 ± 4 nM	-	5.92 ± 0.69 nM	5.78 ± 0.3	8.05 ± 0.5		313.08 ± 2.96
**87**	79 ± 0.3 nM	58 ± 0.3	10.12 ± 0.29 nM	16.13 ± 1.2	22.57 ± 1.5	-	148.321.22
**88**	0.046 ± 0.007 µM	-	0.059 ± 0.009 µM	18.15 ± 1.4	25.39 ± 1.7	72.46 ± 3.6	-
Reference: Sorafenib	-	-	-	7.64 ± 0.4	7.26 ± 0.3	-	77.57 ± 0.57
Reference: Erlotinib	-	-	7.74 ± 0. 59 nM and 0.027 µM	-	-	-	-
Reference: AAZ	86 ± 0.5 µM and 0.039 ± 0.005 µM	33 ± 0.2	-	7.64 ± 0.4	7.26 ± 0.3	-	77.57 ± 0.57
Reference: DOX	-	-	-	3.09 ± 0.1	4.22 ± 0.2	6.67 ± 0.5	-

**Table 17:** Bioassay results for compounds **89**–**97** and reference compounds [65].

Compound	hCA I K_I_ (nM)	hCA II K_I_ (nM)	hCA IX K_I_ (nM)	hCA XII K_I_ (nM)	SI_I/IX_	EGFR IC_50_ (nM)
**8** **9**	71,750	4571	4225	904.1	16.9	132.4 ± 2.4
**90**	78,330	4656	4256	830.8	18.4	82.7 ± 1.7
**91**	2297	435.9	125.4	7.6	18.3	98.3 ± 1.47
**92**	6580	224.1	389.8	35.2	16.9	143.4 ± 2.1
**93**	7602	912.2	404.2	30.8	18.8	84.4 ± 1.25
**94**	6997	257.7	44.0	41.6	159	211.9 ± 7.3
**95**	3891	93.8	38.4	8.9	101.3	51.2 ± 0.97
**96**	2292	396.8	183.4	15.9	12.5	124.3 ± 1.43
**97**	4785	201.8	78.4	11.2	61	128.6 ± 1.2
Reference: AAZ	250.0	12.0	25.0	5.7	10.0	-
Reference: Erlotinib	-	-	-	-	-	80 ± 2.0

**Table 18:** Bioassay results for compounds **98**–**100** and reference compounds [66].

Compound	hCA I K_I_ (µM)	hCA II K_I_ (µM)	hCA IX K_I_ (µM)	EGFR IC_50_ (nM)	MDA-MB-231 IC_50_ (µM)	HeLa IC_50_ (µM)
**9** **8**	>100	>100	>100	95.1 ± 2.8	0.19 ± 0.01	0.14 ± 0.01
**9** **9**	>100	>100	>100	113.0 ± 7.5	0.22 ± 0.01	0.16 ± 0.01
**100**	>100	>100	>100	104.2 ± 4.1	0.13 ± 0.01	0.12 ± 0.01
Reference: AAZ	0.25	0.012	0.026	-	-	-
Reference: Erlotinib	-	-	-	78.6 ± 2.4	-	-
Reference: Staurosporine	-	-	-	-	0.21 ± 0.01	0.39 ± 0.02
Reference: 5-FU	-	-	-	-	0.49 ± 0.02	0.52 ± 0.03

**Table 19:** Bioassay results for compounds **101**–**104** and reference compounds [67].

Compound	hCA II IC_50_ (nM)	hCA IX IC_50_ (nM)	SI	EGFR_WT_ IC_50_ (nM)	EGFR_T790M_ IC_50_ (nM)	A549 IC_50_ (μM)	A431 IC_50_ (μM)	H1975 IC_50_ (μM)
**101**	355.8 ± 61.1	224.3 ± 45.5	1.6	13.7 ± 4.0	37.5 ± 8.2	9.27 ± 0.94	3.91 ± 0.23	3.22 ± 0.27
**102**	278.2 ± 31.6	115.0 ± 16.8	2.4	27.0 ± 6.8	9.2 ± 2.1	6.54 ± 0.59	4.04 ± 0.34	1.94 ± 0.14
**103**	526.2 ± 88.3	577.5 ± 94.6	0.9	51.2 ± 10.4	135.0 ± 15.2	10.14 ± 0.24	21.68 ± 0.16	7.59 ± 0.21
**10** **4**	241.5 ± 43.7	312.8 ± 55.7	0.8	42.6 ± 8.5	93.4 ± 9.8	9.05 ± 0.31	16.76 ± 0.13	4.25 ± 0.21
Reference: AAZ	45.1 ± 7.4	87.2 ± 9.6	0.5	-	-	-	-	-
Reference: Gefitinib	-	-	-	17.1 ± 4.2	378.4 ± 56.8	15.59 ± 1.03	8.37 ± 0.46	10.78 ± 0.45
Reference: Erlotinib	-	-	-	-	-	16.43 ± 0.96	11.85 ± 0.69	13.12 ± 0.97
Reference: Osimertinib	-	-	-	58.2 ± 12.6	8.1 ± 2.2	-	5.32 ± 0.43	0.98 ± 0.01

**Table 20:** Inhibitory activities and CA IX SRs of the most promising compounds.

Compound	hCA IX Inhibition (K_I_ and IC_50_)	SR	VEGFR-2 Inhibition (IC_50_)	EGFR (IC_50_)
**2**	0.077 ± 0.01 μΜ	-	0.069 ± 0.01 μΜ	-
**3**	35 nM	0.21 _**(I/IX)**_	0.058 ± 0.001 μΜ	-
**9**	27.30 ± 1.10 nM	6.69 **_(II/IX)_**	0.06 ± 0.004 μΜ	-
**14**	0.035 ± 0.001 μΜ	-	0.093 ± 0.005 μΜ	-
**2** **3**	3.1 μΜ	24.7 **_(II/IX)_**	32.1 ± 1.10 μΜ	-
**2** **8**	40 nM	-	0.38 ± 0.14 μΜ	-
**4** **7**	8.3 nM	38.76 **_(I/IX)_**	26.3 ± 0.4 nM	-
**6** **5**	3.6 nM	-	204 ± 9 nM	-
**8** **5**	0.039 μM	-	-	0.05 μM
**90**	4256 nM	18.4 **_(I/IX)_**	-	82.7 ± 1.7 nM
**93**	404.2 nM	18.8 **_(I/IX)_**	-	84.4 ± 1.25 nM
**95**	38.4 nM	101.3 **_(I/IX)_**	-	51.2 ± 0.97 nM
**102**	115.0 ± 16.8 nM	2.4 **_(II/IX)_**	-	27.0 ± 6.8 nM (EGFR_WT_) and 9.2 ± 2.1 nM (EGFR_T790M_)

## Data Availability

This article is a review of previously published studies. Data sharing is not applicable to this article.

## Abbreviations

The following abbreviations are used in this manuscript:

5-FU	5-fluorouracil
AAZ	Acetazolamide
AREG	Amphiregulin
CA	Carbonic anhydrase
DOX	Doxorubicin
EGFR	Epidermal Growth Factor Receptor
EGF	Epidermal Growth Factor
EREG	Epiregulin
EC	Extracellular domain
HB-EGF	Heparin-binding EGF-like growth factor
hCA	Human carbonic anhydrase
HIF	Hypoxia inducible factor
HRE	Hypoxia-responsive elements
IC	Intracellular
NSCLC	Non–small cell lung cancer
PlGF	Placental growth factor
PG	Proteoglycan-like domain
RTKIs	Receptor tyrosine kinase inhibitors
SAR	Structure activity relationship
SI	Selectivity index
SR	Selectivity ratio
TM	Transmembrane
TGFα	Transforming growth factor alpha
TK	Tyrosine kinase
RTKIs	Tyrosine kinase inhibitors
VEGF	Vascular endothelial growth factor
VEGFR	Vascular endothelial growth factor receptor
